# Panoramic description of ROS-based nanotechnology for osteomyelitis therapy: Challenges, opportunities, and prospects

**DOI:** 10.7150/thno.124716

**Published:** 2026-02-11

**Authors:** Wenqiao Wang, Xiaoying Kong, Jinsheng Shi, Ting Wang

**Affiliations:** 1 Department of Spine Surgery, The Affiliated Hospital of Qingdao University, Qingdao 266700, China.; 2 Institute of Regenerative Medicine and Laboratory Technology Innovation, Qingdao University, Qingdao 266071, China.; 3 Qingdao Key Lab of Common Diseases, University of Health and Rehabilitation Sciences, Qingdao Municipal Hospital, Qingdao 266071, China.

**Keywords:** osteomyelitis, reactive oxygen species, nanomaterials, nanotechnology

## Abstract

Osteomyelitis, an inflammatory disease of bone and bone marrow caused by infectious microorganisms, has long been a major clinical challenge due to the lack of consistently effective treatment strategies. Conventional therapeutic approaches, such as antibiotic therapy and surgical debridement, are frequently associated with the development of antibiotic resistance and a high risk of disease recurrence, thereby complicating long-term clinical management. In recent years, reactive oxygen species (ROS)-based nanotechnology has emerged as a promising therapeutic modality for osteomyelitis, garnering considerable attention for the potential to overcome antibiotic resistance. This review summarizes the epidemiological characteristics, current treatment approaches, and pathogenic mechanisms of osteomyelitis, and comprehensively examines advances in ROS nanotechnologies for osteomyelitis treatment. In addition, the technical advantages and limitations of major ROS-based strategies, including photodynamic therapy (PDT), sonodynamic therapy (SDT), chemodynamic therapy (CDT), and microwave dynamic therapy (MWDT), are systematically discussed to provide guidance for further optimization of ROS-mediated strategies. Furthermore, the therapeutic potential of these strategies in antimicrobial activity, promotion of tissue repair, and immune regulation is analyzed, offering theoretical support for the integration of ROS-based strategies with existing treatment modalities for improved management of osteomyelitis.

## Introduction

Osteomyelitis is widely recognized as an inflammatory disease of bone and bone marrow caused by infectious microorganisms and is closely associated with local bone destruction, necrosis, and impaired new bone formation 1. The reported annual incidence of osteomyelitis ranges from approximately 4.8 to 21.8 cases per 100,000 population 2. Considerable variation in incidence exists across different populations and geographic regions, largely influenced by factors such as age distribution, underlying comorbidities, prevalence of diabetes, frequency of post-traumatic and post-operative infections, and regional sanitary conditions 3. Although the incidence of acute osteomyelitis has declined in many developed countries as a result of improvements in public health measures and nutritional support, higher rates continue to be observed in several developing regions, particularly in parts of Africa, where urbanization and increased motor vehicle use have contributed to a rising number of open injuries 4. Fracture internal fixation and joint replacement represent common sources of infection leading to osteomyelitis, while elderly individuals with diabetes, neurological disorders, or peripheral vascular disease are at increased risk of developing chronic disease, with approximately 20% of patients with diabetic foot ulcers progressing to osteomyelitis 5. Despite advances in medical care that have reduced the occurrence of acute infection, the incidence of chronic osteomyelitis continues to increase as a consequence of population aging, a growing number of high-energy traumatic injuries, and the expanding volume of orthopedic surgical procedures 6. Bone infection following open fractures and periprosthetic joint infection after arthroplasty often progress to chronic osteomyelitis, imposing a substantial financial burden on affected individuals due to prolonged hospitalization and complex treatment requirements 7. In severe cases, treatment failure may ultimately result in limb amputation or mortality. Consequently, the effective eradication of infection and prevention of disease recurrence have emerged as critical research priorities in the contemporary management of chronic osteomyelitis.

At present, clinically accepted treatment for osteomyelitis remains largely dependent on antibiotic therapy and surgical intervention. Excessive and prolonged use of antibiotics has led to the rapid emergence of antimicrobial resistance and the increasing prevalence of multidrug-resistant bacterial infections. Although surgical debridement enables prompt removal of necrotic and infected tissue, complete eradication of resistant microorganisms remains difficult, thereby increasing the likelihood of disease recurrence 8. Extensive efforts have therefore been directed towards addressing antibiotic overuse and recurrent infection in osteomyelitis management, including the development of novel antimicrobial agents and improvements in surgical techniques and diagnostic strategies; however, clinical outcomes remain suboptimal 9. With recent advances in nanomedicine, reactive oxygen species (ROS)-based nanotechnology has emerged as a promising therapeutic approach for chronic osteomyelitis, demonstrating broad-spectrum antimicrobial activity and effective suppression of drug-resistant and multi drug-resistant pathogens 10.

**Figure 1:**
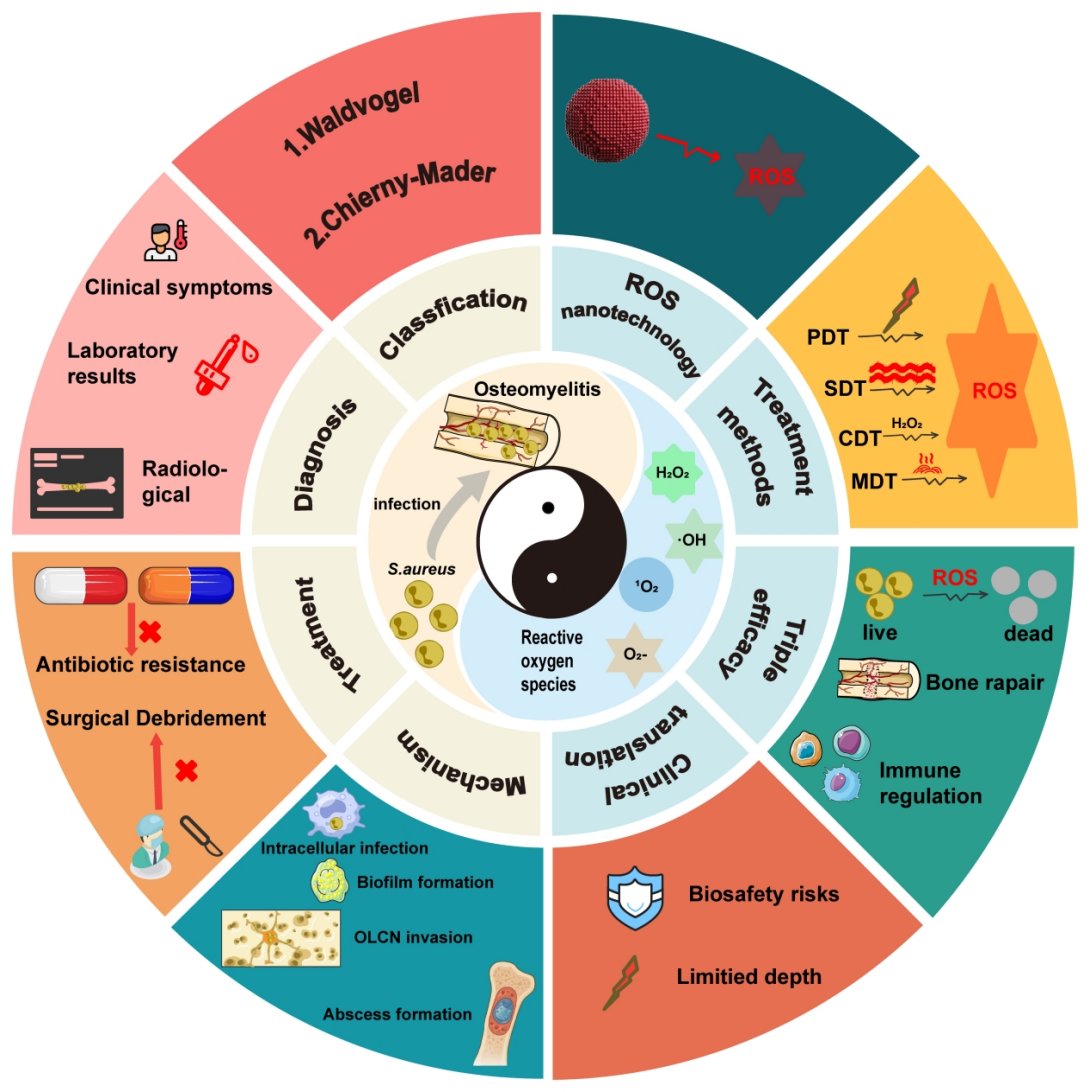
Schematic illustration of the application of reactive oxygen species-based nanotechnology in the treatment of osteomyelitis. The figure summarizes the principal components addressed in this review, including the pathological microenvironment of osteomyelitis, major ROS-mediated therapeutic strategies (PDT, SDT, CDT, and MWDT), and their core functions in antibacterial activity, immune regulation, and bone regeneration.

This review provides a comprehensive and systematic discussion of the clinical classification, pathogenesis, diagnostic techniques, and current treatment strategies for osteomyelitis. Particular emphasis is placed on the mechanisms by which *Staphylococcus aureus* (*S. aureus*), one of the primary causative agents, facilitates persistent infection and immune evasion at the site of infection, thereby contributing to refractory chronic osteomyelitis. Recent progress in ROS-based nanotechnologies for osteomyelitis treatment is subsequently examined in detail. The technical advantages and inherent limitations of major ROS-mediated strategies, including photodynamic therapy (PDT), sonodynamic therapy (SDT), chemodynamic therapy (CDT), and microwave dynamic therapy (MWDT), are analyzed to provide direction for further optimization. In addition, the therapeutic potential of these approaches in antimicrobial activity, enhancement of tissue repair, and modulation of immune responses is evaluated, offering theoretical support for the integration of ROS-based strategies with existing treatment modalities. Finally, current challenges and future prospects of ROS-based nanotechnology in osteomyelitis therapy are discussed to provide a comprehensive overview of emerging treatment strategies and their potential clinical significance (**Figure 1**).

Although recent studies have reported the application of ROS-based technologies in the treatment of bone infections, optimization strategies and combination approaches specifically designed for osteomyelitis, together with clearly defined therapeutic mechanisms, remain insufficiently summarized. Furthermore, the development of ROS technologies tailored to the unique pathological microenvironment of osteomyelitis has received limited attention. Within this context, the present review contributes in three major aspects. First, a comprehensive and critical panoramic analysis of ROS nanotechnologies, including PDT, SDT, CDT, and MWDT, is provided, with particular emphasis on multimodal integration strategies to overcome the limitations of single-modality approaches. Second, beyond the direct antimicrobial effects mediated through disruption of microbial cellular structures, the pivotal role of ROS-based approaches in modulating the immunosuppressive bone microenvironment and their synergistic interactions with bone regeneration strategies are systematically elucidated, leading to the proposal of an integrated triple-therapy framework encompassing anti-infection, immune-regulation, and bone-repair as a comprehensive paradigm for osteomyelitis management. Third, critical challenges and future directions for the clinical translation of ROS nanotechnologies are thoughtfully examined, including considerations of material biosafety, targeting efficiency, rational combination therapy design, and translational feasibility. Collectively, this review provides timely and distinctive guidance for researchers and clinicians pursuing innovative therapeutic strategies for osteomyelitis beyond conventional antibacterial approaches.

## Osteomyelitis

### Pathogenic mechanism of *S. aureus* in osteomyelitis

Owing to the remarkable capacity for invasion, colonization, and proliferation within bone tissue, *S. aureus* exhibits distinct pathogenic behavior during skeletal infection. The major mechanisms underlying persistent osteomyelitis include intracellular infection, formation of staphylococcal abscess communities (SACs), invasion of the osteocyte lacuno-canalicular network (OLCN), and biofilm formation 11. Systematic analysis of these mechanisms not only clarifies the specific pathway by which *S. aureus* invades and colonizes bone tissue but also provides a conceptual foundation for the development of novel therapeutic strategies for refractory osteomyelitis.

**Figure 2:**
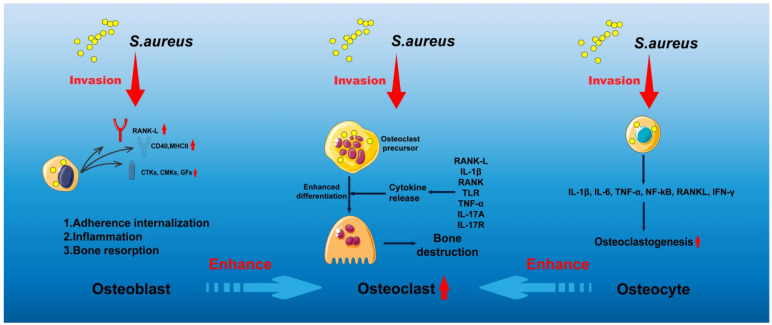
Summary diagram illustrating the responses of osteoblasts, osteoclasts, and osteocytes in the presence of S. aureus.

**Intracellular infection:** Intracellular infection represents a central mechanism contributing to the persistence and recurrence of osteomyelitis, as *S. aureus* is capable of long-term survival in a dormant state within host cells. Multiple cell types, including macrophages, endothelial cells, keratinocytes, and epithelial cells, have been shown to harbor intracellular *S. aureus* 12-14. Infection of macrophages is particularly associated with chronic osteomyelitis. These infected macrophages, often described as "Trojan horse" macrophages, facilitate systemic bacterial dissemination, promote the enrichment of small colony variants (SCV), and attenuate host inflammatory responses 14. Such phenotypic adaptation enables *S. aureus* to evade immune surveillance and constitutes a major driver of chronic and recurrent infection. In addition, *S. aureus* is capable of invading osteoblasts, osteoclasts, and osteocytes (**Figure 2**), where intracellular persistence eventually leads to host cell apoptosis or sustained intracellular infection, further perpetuating disease progression 15-26.

**Formation of SACs:** *S. aureus* can achieve effective self-protection within the host microenvironment through the formation of specialized protective barriers, gradually developing into SACs that inhibit immune cell attack and facilitate bacterial survival and dissemination 27. SACs, representing abscess structures associated with low- virulence bacterial infection, are mainly found within the bone marrow cavity of the long bones in pediatric patients. Structurally, SACs consist of a central bacterial community surrounded a fibrin-rich pseudocapsule and infiltrating immune cells, a configuration that confers high bacterial viability and substantial resistance to both antibiotic treatment and host immune defenses 28. SAC formation is governed by complex regulatory signaling pathways. Initially, *S. aureus* activates coagulase (CoA) and von Willebrand factor-binding protein (vWFbp), which bind to and activate prothrombin, thereby promoting the formation of protective fibrin pseudocapsules 29. Subsequently, clumping factor A (ClfA) and clumping factor B (ClfB) interact with fibrinogen, enabling the pathogen to evade phagocytic recognition and contributing critically to SAC development 30. In parallel,* S. aureus* secretes immune evasion proteins, such as Staphylococcal chemoattractant proteins (CHIPS) and Staphylococcal complement inhibitors (SCIN), which suppress acute immune responses 31. Furthermore, *S. aureus* induces destruction of neutrophils recruited to the abscess community, generating cellular debris that paradoxically forms an additional physical barrier, thereby preventing subsequent infiltration of host defense cells and further enhancing bacterial persistence 32.

**Formation of Biofilm:** Biofilms are organized microbial communities that adhere to tissue surfaces or biomaterial interfaces and represent one of the most prevalent complications in orthopedic infections. The surfaces of commonly used orthopedic materials, such as titanium and its alloys, stainless steel, cobalt-chromium alloys, polymeric materials, and polymethyl methacrylate (PMMA) bone cement, provide favorable substrates for biofilm development. Biofilm formation by *S. aureus* at sites of bone injury significantly restricts the penetration of antibiotics and immune cells, thereby promoting persistent infection 33. Biofilm development generally proceeds through sequential stages of bacterial attachment, accumulation, and dispersion within host tissues 34-35. Bacteria released from mature biofilms secrete a broad range of virulence factors, including coagulase, lipase, hyaluronidase, and protein A (SpA), which play important roles in disease progression and osteomyelitis pathogenesis. Among these, toxic shock syndrome toxin-1 (TSST-1), alpha-hemolysin (Hla), and Pantone-Valentine leukocidin (PVL) have been shown to significantly exacerbate osteomyelitis. TSST-1, a potent superantigen that suppresses plasma cell differentiation and antibody responsiveness, markedly inhibits host immune function and induces elevated expression of cytokines associated with toxic shock syndrome, including IFN-γ, TNF, IL-2, and IL-6 36. Furthermore, TSST-1 has been demonstrated to enhance osteoclast-mediated bone resorption and accelerate bone tissue destruction 37. Hla, a secreted cytotoxin synthesized by many *S. aureus* strains, disrupts early neutrophil recruitment by impairing calcium signaling and calcium-dependent leukotriene B4 (LTB4) production 38; however, Hla expression is downregulated during persistent bone infection, contributing to bacterial quiescence and the latent nature of recurrent osteomyelitis 39. PVL, produced by a limited subset of *S. aureus* strains, lyses leukocytes through membrane disruption and is strongly associated with rapid disease progression and sustained infection 40. In addition, *S. aureus*-derived coagulase promotes biofilm stabilization by converting fibrinogen into fibrin, thereby generating a protective barrier that shields bacteria from immune attack. Coagulase further contributes to bone destruction by inhibiting osteoblast proliferation, inducing apoptosis, and reducing mineralization, ultimately aggravating bone loss during infection 41.

**OLCN invasion:** Invasion of the OLCN by *S. aureus* represents another critical mechanism underlying the persistence of osteomyelitis, enabling the pathogen to evade host defenses more effectively and substantially increasing the risk of recurrence following treatment of refractory osteomyelitis 11. Emerging evidence indicates that *S. aureus* can penetrate cortical bone and persist within the OLCN, a process attributed in part to the remarkable deformability of the bacterial cells 42. Using transmission electron microscopy (TEM), de Mesy *et al.* directly visualized the invasion and colonization of cortical bone by *S. aureus* and demonstrated that the bacteria are capable of deforming to diameters of approximately 0.5 µm to facilitate migration through sub micron bone channels 43. Consistent with these findings, bacterial deformation and infiltration of the OLCN were subsequently observed in lesion samples from patients with infectious diabetic foot ulcers complicated by chronic *S. aureus* osteomyelitis 44. Notably, clinical reports describing reactivation of infection 50 to 75 years after the initial episode further support the capacity of *S. aureus* for long-term persistence within bone tissue, likely mediated by submicron deformation and slow metabolic activity of *S. aureus* within the osteocellular canicular system 45-47.

### Traditional treatments for osteomyelitis

Effective management of osteomyelitis and prevention of disease recurrence require a comprehensive, interdisciplinary treatment approach encompassing thorough patient evaluation, appropriate antimicrobial therapy, and necessary surgical intervention. Selection of specific treatment modalities is influenced by multiple factors, such as disease pathogenesis, anatomical site, presence of orthopedic implants, and host-pathogen interactions, resulting in significant interindividual variability. Despite these differences, the core objectives of osteomyelitis management remain consistent, namely the eradication of local infection and control of systemic dissemination 48. This section provides a systematic overview of conventional antibiotic-based and surgical treatment strategies, together with an analysis of their inherent limitations, with the aim of informing optimization of current therapeutic approaches and identifying potential directions for future development.

**Antibiotic therapy:** Antibiotic administration remains essential in the treatment of osteomyelitis, regardless of whether surgical debridement is performed 49. Systemic antimicrobial therapy constitutes an important component of disease management, with empirical broad-spectrum antibiotics typically initiated prior to the availability of microbiological culture and sensitivity results. Owing to the high likelihood of rapid resistance development with monotherapy, combination antibiotic regimens are commonly employed when formulating clinical treatment plans 50. Despite widespread clinical use, systemic antibiotic therapy is associated with substantial limitations. Prolonged high-dose exposure frequently increases the risk of multi-organ toxicity, while long-term treatment contributes to the development of irreversible antimicrobial resistance. The clinical application of local antibiotic delivery for osteomyelitis remains controversial 51-54, and existing localized strategies face additional challenges, including suboptimal drug release kinetics, limited biocompatibility, and the need for secondary surgical procedures.

**Surgical Treatment:** Surgical management of osteomyelitis is primarily directed toward thorough debridement of infected tissue, reconstruction of bone and soft tissue defects, and prevention of associated complications. In chronic osteomyelitis, debridement typically involves removal of inflammatory tissue, sinus tracts, scar tissue, infected granulation tissue, medullary abscesses, sclerotic bone, and necrotic bone. Clinical outcomes following surgical intervention are strongly influenced by anatomical location, integrity of surrounding soft tissues, presence of orthopedic implants, formation of deep abscesses or biofilms, and host immune status, resulting in considerable interpatient variability 55. Osteomyelitis associated with diabetic foot infection presents particular therapeutic challenges, often accompanied by severe soft tissue infection, neuroarthritic osteoarthropathy, and compromised vascular supply, which collectively contribute to suboptimal treatment outcomes 56-57. Consequently, patients with metabolic disorders require highly individualized therapeutic strategies. Moreover, even after technically successful surgical intervention, osteomyelitis remains associated with a substantial risk of recurrence, underscoring the need for adjunctive technologies capable of achieving complete eradication of residual microorganisms while simultaneously promoting restoration of the local microenvironment.

**Other Treatments:** Hyperbaric oxygen therapy has shown unique therapeutic advantages in the treatment of osteomyelitis. The antibacterial effect of hyperbaric oxygen in osteomyelitis is thought to be closely related to enhanced formation of ROS 58. In addition, hyperbaric oxygen therapy reduces localized pathogen burden through activation of host immune responses and synergistic interaction with antimicrobial agents, while its anti-inflammatory properties further contribute to the attenuation of bone tissue injury and infection 59. Beyond oxygen-based approaches, growth factor- and gene-based strategies have also been explored for osteomyelitis treatment. Aliyev *et al.* demonstrated that vascular endothelial growth factor (VEGF) gene-transfected muscle flaps significantly reduced abscess formation and bone necrosis by inhibiting disease progression and promoting local blood supply, supporting the development of molecular therapies for osteomyelitis 60. Moreover, a growing number of emerging technologies have reported considerable potential for the treatment of infectious diseases 61-66, among which ROS-based antimicrobial strategies represent particularly promising therapeutic candidates 67.

## Application of ROS nanotechnology in osteomyelitis

In recent years, ROS-based antibacterial nanotechnology has attracted increasing attention in the clinical management of osteomyelitis due to its potent antimicrobial efficacy and low propensity for inducing drug resistance. The core mechanism underlying ROS-mediated antibacterial nanotechnology involves exploitation of the physicochemical properties of nanomaterials to generate ROS, thereby disrupting the redox homeostasis of microbial cells and ultimately inducing cell death. This section systematically examines the potential advantages and therapeutic applications of ROS-based antibacterial nanotechnology in osteomyelitis treatment, with the aim of providing valuable insights for the development of next-generation anti-osteomyelitis therapies.

### Overview of ROS

ROS are highly reactive and unstable oxygen-derived molecules generated as byproducts of cellular oxygen metabolism, primarily within organelles such as mitochondria, endoplasmic reticulum, and peroxisomes. Major ROS species include free radicals such as superoxide anion (·O_2_^-^), hydroxyl radical (·OH), hydroperoxyl radical (·HOO), carbonate radical (·CO_3_^-^), and carbon dioxide radical (·CO_2_^-^), as well as non-radical oxidants including singlet oxygen (^1^O_2_), hydrogen peroxide (H_2_O_2_), hypobromous acid (HOBr), and hypochlorous acid (HOCl) 68.

Acting as critical intracellular signaling mediators, ROS regulate multiple canonical signaling pathways involved in inflammation and infection. ROS activate upstream kinases of the mitogen-activated protein kinase (MAPK) cascade, including c-Jun N-terminal kinase (JNK) and extracellular regulatory protein kinase (ERK), thereby promoting activation of the transcription factor AP-1 and inducing expression of genes related to cell proliferation, survival and inflammatory responses 69. Accumulation of ROS further amplifies phosphatidylinositol 3-kinase/protein kinase B (PI3K/Akt) signaling, thereby enhancing downstream pathways that support cellular survival and proliferation. Sustained ROS production also maintains continuous activation of the JAK/STAT pathway by stimulating Janus kinases and inhibiting protein phosphatases such as SHP-2, ultimately driving transcription of inflammation-related genes 69. Within the nuclear factor κB (NF-κB) signaling network, ROS often act as second messengers, oxidizing NF-κB directly or modifying key cysteine residues on IκB, thereby promoting dissociation of the NF-κB-IκB complex and promoting NF-κB nuclear translocation, which culminates in the expression of pro-inflammatory cytokines 70. In parallel, ROS modulate the antioxidant response through activation of the Nrf2 pathway. Oxidative modification of key cysteine residues on Keap1 leads to Nrf2 release and nuclear translocation, thereby initiating the transcription of antioxidant enzyme, including heme oxygenase-1 (HO-1) and NAD(P)H quinone dehydrogenase 1 (NQO1), to counterbalance oxidative stress. Dysregulation of these interconnected pathways has been observed in chronic inflammatory and infectious conditions, where excessive ROS simultaneously hyperactivate pro-inflammatory cascades such as MAPK, NF-κB, and PI3K/Akt while suppressing Nrf2-mediated antioxidant defenses, thereby driving amplification of tissue-destructive inflammatory responses 69.

Substantial endogenous production of ROS at sites of infection induces oxidative stress and triggers amplification of pro-inflammatory signaling pathways, including MAPK, NF-κB, and NLRP3, thereby reshaping the local microenvironment in osteomyelitis 69,70. This pathological microenvironment is characterized by persistent inflammation, enhanced cellular apoptosis, and increased bone resorption. The elevated ROS levels within infected bone tissue provide valuable molecular targets for diagnostic applications. For instance, ROS-responsive fluorescent and photoacoustic probes can be selectively "illuminated" within inflamed bone tissues, aiding early lesion localization and dynamic monitoring of disease progression.

Osteomyelitis lesions are further distinguished by persistent microbial colonization and biofilm formation accompanied by immune suppression or dysfunction. ROS exert direct antimicrobial effects by inducing lipid peroxidation and damaging microbial proteins and nucleic acids, thereby serving as a non-antibiotic, broad-spectrum antimicrobial mechanism. Concurrently, physiologically moderate ROS signaling modulates immune responses by activating or reprogramming immune cells. Experimental evidence indicates that ROS promote macrophage polarization toward the M1 phenotype, enhancing bactericidal activity, and enhancing adaptive immune responses through regulation of T-cell activation.

At low to moderate concentrations, ROS promote angiogenesis, osteoblast proliferation and differentiation, and tissue regeneration. In contrast, elevated ROS levels exhibit superior antimicrobial efficacy at infected bone sites but may also induce collateral bone damage. To mitigate ROS-associated osteotoxicity following antibacterial treatment, integrated therapeutic strategies combining ROS-based antimicrobial approaches with osteogenic modalities have been developed, including growth factor delivery, osteoconductive scaffolds, and bioactive ion release systems. These combinatorial platforms enable precise antimicrobial action while simultaneously promoting bone defect repair and reconstruction of the pathological bone microenvironment.

### Classification of ROS nanotechnology

Recent advances in nanotechnology, particularly in nanochemistry and nanofabrication, have enabled the development of a diverse range of ROS-generating nanomaterials with microenvironment-responsive and regulatory properties, which are increasingly applied across biomedical fields, including osteomyelitis therapy 71. These nanomaterials produce distinct ROS upon stimulation by near-infrared (NIR), ultrasound (US), chemical reaction substrate, microwave energy, and related triggers, ultimately inducing irreversible bacterial damage and apoptosis. On the basis of their mechanisms of ROS generation and associated energy sources, ROS-based nanotechnologies are broadly categorized into PDT, SDT, CDT, and MWDT 72 (**Table 1**).

PDT employs photosensitizers that generate ROS upon irradiation with specific wavelengths of light, thereby directly inactivating pathogenic microorganisms. PDT exhibits broad applicability and a low propensity for inducing drug resistance. Within the hypoxic microenvironment characteristic of osteomyelitis, PDT predominantly generates ·O_2_^-^ and ·OH through electron transfer-mediated Type I photochemical reactions, enhancing antimicrobial efficacy against drug-resistant bacteria.

SDT utilizes deep-penetrating ultrasound to excite sonosensitizers, leading to ROS production and mechanical effects. SDT demonstrates favorable penetration and targeting properties for deep-seated bone marrow infections while exerting minimal impact on surrounding healthy tissues. Furthermore, cavitation and shear forces generated during SDT disrupt bacterial biofilm architecture, thereby improving antibacterial efficiency. CDT exploits nanozyme-mediated Fenton or Fenton-like reactions within infection tissues to catalyze the conversion of endogenous hydrogen peroxide (H_2_O_2_) into highly reactive ·OH, enabling sustained ROS generation for antimicrobial activity without external energy input. In addition, certain catalytic systems incorporating calcium and magnesium release bioactive metal ions during the reaction process, synergistically promoting bone regeneration. MWDT relies on microwave sensitizers that generate both thermal effects and ROS under microwave irradiation, offering advantages of deep tissue penetration and highly efficient sterilization.

### PDT for osteomyelitis treatment

#### Overview of PDT in osteomyelitis treatment

In the early twentieth century, von Tappeiner and Rabb first proposed the potential therapeutic potential of photosensitive synthetic dyes in combination with light and molecular oxygen and introduced the concept of "photodynamic action" 73. Recently, PDT-based medical devices have been developed and approved for clinical application by the U.S. Food and Drug Administration (FDA), underscoring the growing translational relevance of this modality 74,75.

PDT relies on the synergistic interaction among photosensitizers, tissue oxygen concentration, and an appropriate light source to induce microbial inactivation. Unlike conventional antimicrobial agents, PDT triggers cytotoxic reactions through light-induced excitation of photosensitizers, resulting in the generation of ROS that ultimately disrupt bacterial structures and functions. This mechanism represents a distinct class of anti-infective therapy with demonstrated efficacy against drug-resistant pathogens in both *in vivo* and *in vitro* settings. On this basis, photodynamic antimicrobial chemotherapy (PACT) has been developed as a specialized application of PDT. PACT is founded on the selective location of non-toxic photosensitizers within pathogenic microorganisms, thereby minimizing off-target toxicity to surrounding tissues, followed by light activation at specific wavelength to generate highly cytotoxic ROS that inactivate pathogens 76. PACT is therefore particularly suited for the treatment of microbial infections, including bacterial, fungal, viral, and parasitic diseases. In the context of osteomyelitis therapy, photosensitizers are delivered to the infected bone marrow cavity through systemic administration, such as intraperitoneal or intravenous injection, or by local injection. After a defined dark incubation period, the photosensitizer accumulates preferentially at the site of infection via blood circulation. Subsequent laser irradiation of the affected region induces localized ROS production, leading to efficient bacterial inactivation within the bone marrow cavity and therapeutic resolution of infection. Subsequent studies further confirmed the pronounced antibacterial efficacy of PACT in the bone marrow infections 77. Notably, the successful application of PACT for the treatment of diabetic foot-associated osteomyelitis reported by Tardivo *et al.* demonstrated the safety and clinical effectiveness of this approach, offering a promising therapeutic option for refractory osteomyelitis 78.

#### Development and innovation of PDT for osteomyelitis treatment

##### Development of photosensitizers for osteomyelitis treatment

An ideal photosensitizer should exhibit high photodynamic efficiency, low intrinsic toxicity, adequate water solubility, and strong targeting capability 75. Currently, photosensitizers used in PDT for osteomyelitis mainly include 5-aminolevulinic acid (5-ALA) and phenothiazine derivatives. Conventional phenothiazine compounds, such as methylene blue and toluidine blue O (TBO), function as cationic photosensitizers and demonstrate favorable photodynamic antibacterial activity 75. An *in vitro* study investigating the inhibitory effects of PDT on mature methicillin-resistant *Staphylococcus aureus* (MRSA) biofilms *in vitro* revealed that TBO-mediated PDT induced pronounced morphological alterations, including biofilm shrinkage, fissuring, fragmentation, and thinning, accompanied by significant suppression of bacterial virulence 79.

Despite these advantages, clinical application of non-endogenous photosensitizers in antimicrobial therapy remains constrained by limitations related to incomplete characterization of metabolic pathways and potential cytotoxicity. In contrast, 5-ALA is rapidly metabolized into protoporphyrin IX by ALA dehydratase *in vivo*, resulting in reduced effective concentrations and heterogenous distribution, which in turn compromise PDT efficiency. Consequently, development of novel photosensitizers specifically tailored for osteomyelitis therapy represents an important task for advancing PDT-based treatment strategies. Yin *et al.* designed a new cationic photosensitizer, LD4, and evaluated its therapeutic performance in a rabbit tibial acute osteomyelitis model. *In vivo* results showed that LD4 exhibits good water solubility, low toxicity, targeting, strong targeting capacity, and pronounced antimicrobial activity in the treatment of infectious diseases 80. Compared with conventional photosensitizers, LD4 exhibited broader photoinactivation efficacy as well as improved biocompatibility and physicochemical stability. Moreover, LD4-mediated PDT showed potential benefits in promoting bone healing and alleviating bone defects. Nevertheless, comprehensive assessment of potential adverse effects and long-term therapeutic outcomes of LD4 requires further validation through extended follow-up and clinical investigation.

##### Development of PDT nanomaterials for osteomyelitis therapy

**Figure 3:**
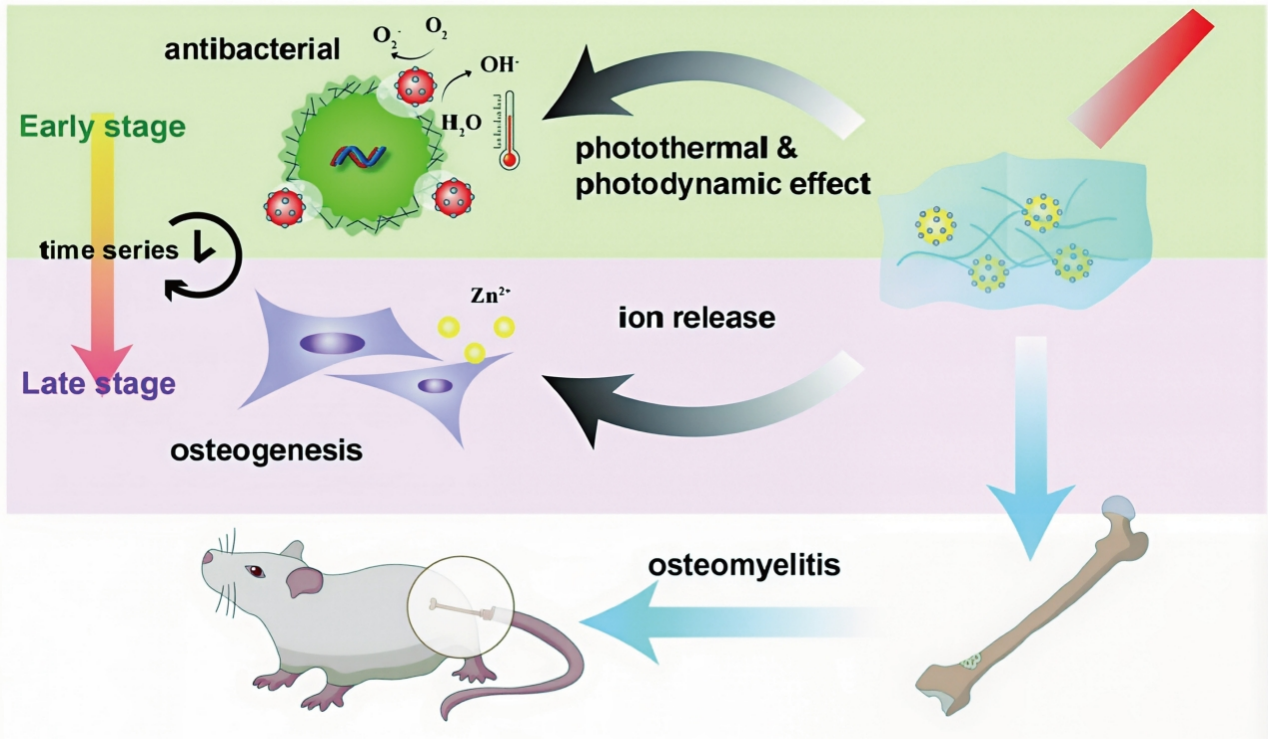
Schematic illustration of ZnO/Ag_2_S nanoparticles exhibiting combined photothermal and photodynamic effects for osteomyelitis treatment. Adapted with permission from Ref. 83. Copyright 2023, American Chemical Society.

At present, photosensitizer-mediated PDT, which has been successfully applied in the clinical treatment of tumors and superficial skin infections, has not yet been widely implemented for osteomyelitis management, primarily due to the mismatch between the deep anatomical location of osteomyelitis lesions and the limited issue penetration of conventional light sources. Enhancement of light penetration depth is therefore crucial for extending PDT applicability to osteomyelitis. The wavelength and energy density of the irradiation source exerts decisive influence on PDT efficacy, while tissue density and thickness further modulate penetration depth. NIR light exhibits superior tissue penetration compared with ultraviolet and visible wavelengths. Salehpour *et al.* reported that whereas the transmittance of a 660 nm laser through the scalp and skull was approximately 5.8%, an 810 nm laser achieved transmittance of (51.41 ± 2.12)% through the skull of male rats 81. Notzli *et al.* further demonstrated that the maximum NIR penetration depth in cortical bone, cartilage, and trabecular bone reached 2.9 ± 0.2 mm, 3.5 ± 0.3 mm, and 3.5 ± 0.2 mm, respectively, confirming the feasibility of NIR-mediated PDT for osteomyelitis treatment 82. Building upon these findings, multiple studies have explored NIR-activated PDT platforms for osteomyelitis therapy. For example, Wu *et al.* designed a biocompatible core-shell nanomaterial, ZnO/Ag_2_S nanoparticles, in which incorporation of Ag_2_S optimized the band-gap structure of ZnO, thereby improving photoelectric efficiency and ROS generation capacity. Density functional theory and ultraviolet photoelectron spectroscopy confirmed the stable photothermal and photodynamic properties of ZnO/Ag_2_S nanoparticles. These nanomaterials showed potent antibacterial activity upon NIR activation during the acute phase of infection and subsequently released Zn^2+^ ions during the chronic phase to promote bone regeneration 83 (**Figure 3**).

##### Combination of multimodal imaging and PDT in osteomyelitis therapy

In recent years, the combination of PDT with advanced imaging techniques, such as photoacoustic imaging (PAI) and magnetic resonance imaging (MRI), has enabled the development of dual-modal diagnostic and therapeutic platforms for osteomyelitis 84,85. Compared with conventional optical imaging techniques, PAI offers the advantages of high optical contrast, superior US-based spatial resolution, and increased tissue penetration depth 86. MRI, in contrast, provides excellent soft-tissue contrast and spatial resolution without the risks associated with ionizing radiation. Notably, PAI compensates for the relatively low spatial resolution of MRI, while MRI offsets the limited penetration depth of PAI. Consequently, the combined application of PAI and MRI during PDT treatment enables accurate delineation of infected regions and real-time guidance of antibacterial therapy, while overcoming the inherent limitations of single-modality imaging. PAI/MRI dual-modal imaging platforms facilitate comprehensive whole-body assessment alongside high-resolution visualization of local tissue architecture, thereby significantly improving early diagnosis and therapeutic precision in osteomyelitis management. For example, in addressing the clinical challenges of early detection and effective intervention, Lu and colleagues developed a multifunctional theranostic agent integrating indomyanine green-mediated PAI with Mn^2+^-enhanced MRI. This agent selectively accumulated at infection sites and enabled precise lesion identification under both MRI and US imaging, while simultaneously supporting PDT-based therapy. This work represents a successful example of integrated early diagnosis and photodynamic treatment for osteomyelitis 87.

In summary, the spatiotemporal controllability and broad-spectrum antimicrobial activity of PDT render this modality particularly well suited for osteomyelitis treatment. The noninvasive nature of PDT, together with its compatibility with advanced imaging techniques, provides a strong foundation for clinical translation in osteomyelitis treatment.

#### Challenges and prospects of PDT for osteomyelitis therapy

Despite significant progress, application of PDT in osteomyelitis particularly in deep-seated bone infections, remains constrained by limited light penetration, most notably the inability of conventional light sources to effectively traverse dense cortical bone. Although the introduction of NIR and development of advanced photosensitizers have partially alleviated these technical barriers, such strategies simultaneously raise concerns regarding undefined therapeutic dosages and potential biological safety risks. Furthermore, the oxygen dependence of conventional type II PDT restricts its therapeutic efficacy within the hypoxic microenvironment characteristic of chronic bone infection and biofilm-associated lesions. To address these challenges, several research teams have explored Type I photosensitizers and engineered oxygen-generating or oxygen-carrying nanomaterials; however, these approaches substantially increase the complexity of material design and introduce additional safety considerations. Additionally, comprehensive evaluation of the long-term biosafety, pharmacokinetics, and biodistribution of metal-based nanomaterials and organic photosensitizers remains essential prior to widespread clinical application. Therefore, future breakthroughs in PDT for osteomyelitis treatment hinge on the development of deeply penetrating and/or oxygen-independent photo therapeutic systems, such as NIR-II-responsive platforms and highly efficient self-oxygenating nanostructures, in parallel with the establishment of standardized light delivery protocols optimized for bone tissue and rigorous safety evaluation of next-generation photosensitizers.

In summary, PDT exerts antibacterial effects through light-activated photosensitizers that generate ROS, leading to microbial destruction. Nevertheless, reduced efficacy in deep infections, especially in osteomyelitis, remains a principal limitation. In this context, SDT, which offers superior tissue penetration, is anticipated to complement PDT and compensate for its intrinsic shortcomings, thereby playing an increasingly significant role in the treatment of deep bone infections.

### SDT for osteomyelitis therapy

#### Overview of the application of SDT in osteomyelitis therapy

In 1989, Yumita *et al.* first reported the responsiveness of hematoporphyrin to US) irradiation and its associated antitumor effects, thereby introducing the therapeutic concept of SDT 88. As a periodically oscillating mechanical wave, US possesses the capability to penetrate biological tissues to depths of up to approximately 10 cm, supporting the application of SDT in the treatment of deep-seated tumors, deep infections, and other pathological conditions in recent years 89.

The therapeutic mechanisms of SDT involve multiple synergistic processes, including mechanical injury caused by US cavitation, ROS generation mediated by sonosensitizer activation, regulation of intracellular signaling pathways leading to apoptosis, and enhancement of host body immune responses. Among these, US cavitation = and ROS production are considered the principal contributors to the potent antibacterial efficacy of SDT. Cavitation phenomena during SDT are generally classified into non-inertial and inertial cavitation 90. Under conditions of localized high pressure and temperature, US cavitation within liquid environments produces mechanical forces, such as microstreaming, shock-wave generation, and sonoporation, that induce cellular damage and apoptosis. Sonoporation, in particular, results in the transient formation of reversible micropores on the cell membrane ranging from a few nanometers to several hundred nanometers in diameter, thereby increasing membrane permeability and facilitating improved delivery of therapeutic agents, genes, and bioactive molecules. Two main mechanisms have been proposed to explain ROS generation during SDT. In the first, collapse of cavitation bubbles during inertial cavitation triggers sonoluminescence, which stimulates the sonosensitizer and promotes reactions with molecular oxygen to generate ROS within tissues. In the second, localized high temperatures generated during US irradiation of liquid systems promote dissociation of oxygen-containing molecules, leading to ROS formation 91,92.

Compared with PDT, SDT offers superior tissue penetration and improved biosafety due to the mechanical nature of US energy. A variety of acoustic sensitizers, including precious metal-doped TiO_2_ (s.g., Pt, Pb, and Au), porphyrin-based single-atom catalysts, metal-organic framework nanoparticles, and piezoelectric nanomaterials such as MoS_2_ and BaTiO_3_, have exhibited effective SDT activity and the capacity to induce microbial apoptosis 93. In recent years, the application of SDT in bacterial infection has expanded substantially, with particular promising outcomes reported in the treatment of osteomyelitis 94,95^.^

#### Development and innovation of SDT for osteomyelitis treatment

##### Development of acoustic sensitizers for osteomyelitis treatment

Over the past few decades, following the discovery of the acoustic activity of hematoporphyrin, an expanding range of acoustic sensitizers has been developed for antitumor and antibacterial applications. Advances in pharmaceutical nanotechnology have not only improved the stability and targeting efficiency of conventional porphyrin-based sensitizers but have also promoted the exploration of sound-responsive characteristics in various nanomaterials, thereby significantly enriching the application potential of sonosensitizing agents. Currently, acoustic sensitizers are mainly classified into organic sensitizers, inorganic sensitizers, and organic-inorganic hybrid sensitizers 96.

Organic acoustic sensitizers, representing the earliest class of agents applied in SDT, exhibit both photosensitive characteristics and sonodynamic activity under US irradiation. This category primarily consists of porphyrins, phthalocyanines, xanthene derivatives, phenothiazine compounds, fluoroquinolones antibiotics, natural products, and other organic small-molecule sonosensitizers 96. Organic sensitizers are distinguished by high ROS production efficiency, tunable SDT performance, and favorable biodegradability. However, limitations such as poor water solubility, chemical instability, rapid systemic clearance, low bioavailability, and potential phototoxicity have restricted broader clinical application in antimicrobial therapy. In contrast, inorganic sensitizers, represented by TiO_2_, ZnO_2_, Bi_2_MoO_6_, BaTiO_3_, and MnWO_x_, offer superior physicochemical stability and allow flexible regulation of pore structure and surface functionalization, thereby providing notable advantages for *in vivo* targeted delivery 91. Nonetheless, the limited biodegradability of inorganic sensitizers may result in cumulative systemic toxicity and long-term biosafety concerns, underscoring the importance and rigorous safety evaluation.

To address the respective limitations of organic and inorganic acoustic sensitizers, organic-inorganic hybrid acoustic sensitizers that integrate the advantages of both classes have been successfully developed and applied in recent years for exploratory clinical use in SDT. Among these, metal-organic framework (MOF)-based acoustic sensitizers have emerged as a major focus of current research 97. MOF sonosensitizers consist of crystalline structures formed through coordination between metal ions or clusters and organic acoustic-sensitive ligands, featuring well-defined pores and cavities. The controllable cavity dimensions of MOFs enable incorporation of diverse functional components—such as quantum dots, nanoclusters, organic small molecules, or nanoparticles—either during or after synthesis, facilitating construction of multifunctional composite platforms that combine the beneficial properties of organic and inorganic acoustic nanomaterials. In addition, the large specific surface area, adjustable porosity, facile surface modification, and high drug-loading capacity of MOFs provide strong support for their application in antimicrobial SDT. The tunable pore architecture and intrinsic catalytic activity further positioned MOFs as highly promising next-generation sonosensitizers.

##### SDT nanomaterials for osteomyelitis therapy

**Figure 4:**
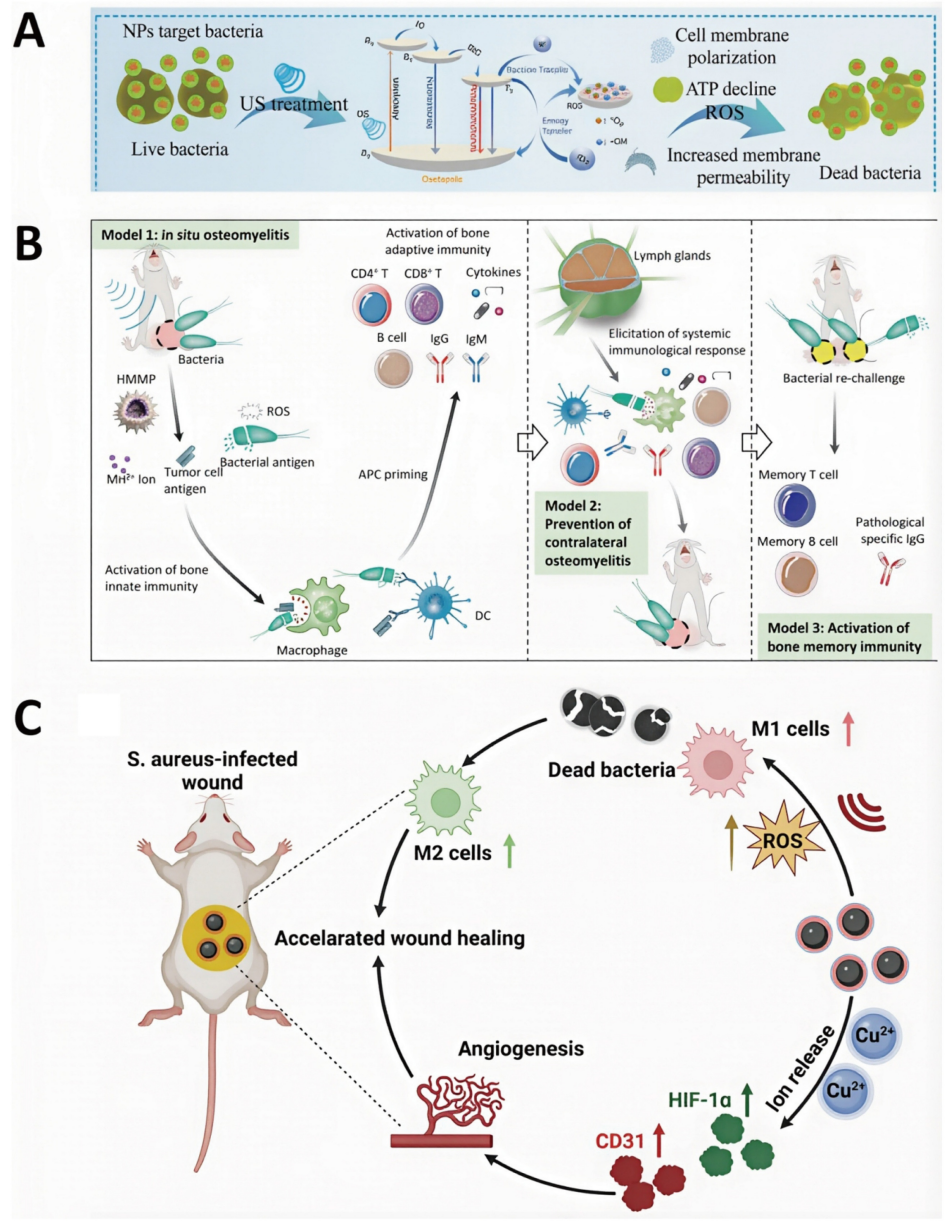
(A) Cationic starch-modified curcumin nanoparticles (CS@Cur NPs) for the treatment of MRSA-induced osteomyelitis, exhibiting anti-inflammatory activity and promotion of osteogenic differentiation. Adapted with permission from Ref. 98. Copyright 2023, Wiley-VCH. (B) Schematic illustration of HMMP construction. Adapted with permission from Ref. 99. Copyright 2022, American Chemical Society. (C) Scheme representation of PtCu-PEG NP preparation for sonodynamic antibacterial therapy and tissue repair. Adapted with permission from Ref. 100. Copyright 2023, Wiley-VCH.

Targeted accumulation of acoustic sensitizers at osteomyelitis lesions is crucial for improving the therapeutic efficacy and bactericidal performance of SDT. For instance, Chen *et al.* developed cationic starch-modified curcumin nanoparticles (CS@Cur NPs). Owing to their positive surface charge, CS@Cur NPs effectively target bacteria with negatively charged cell membranes, resulting in capture rates of 34.7% for *E. coli* and 35.8% for *S. aureus*, respectively. Upon US irradiation, CS@Cur NPs produce substantial levels of ROS, inducing oxidative stress-mediated bacterial death through increased membrane permeability and disruption of ATP synthesis 98 (**Figure 4A**). In addition, biomimetic nanotechnology has also been used to further improve the targeting efficiency of SDT nanomaterials toward infected bone marrow. Lin *et al.* designed a biomimetic nanotherapeutic system, HMMP, constructed by combining hybrid membranes derived from macrophages and tumor cells with hollow MnO_x_ nanoparticles encapsulating protoporphyrin IX (PpIX). This platform enabled efficient targeting of osteomyelitis infection sites and controlled ROS release, resulting in highly effective bactericidal activity within infected bone marrow 99 (**Figure 4B**).

The pathological microenvironment of osteomyelitis is characterized by acidic pH and high GSH levels, and adaptation of nanomaterials to these conditions is critical for improving SDT-mediated therapeutic outcomes. Stability of nanoparticles under acidic conditions represent a key requirement for effective SDT performance. Chen *et al.* investigated the influence of pH on the antibacterial effect of CS@Cur NPs and showed that these nanoparticles maintained stable and potent antimicrobial activity across a pH range of 3-7 98 (**Figure 4A**). To counteract the inhibitory effects of high GSH levels on ROS-mediated antibacterial activity, Cheng *et al.* designed ultrasmall platinum-copper alloy nanoparticles (PtCu-PEG NPs) with strong GSH-depleting ability. Through enhanced ROS-based bactericidal mechanisms, PTKU-PEG NPs effectively suppressed deep *S. aureus*-induced osteomyelitis infection 100 (**Figure 4C**). These findings highlight the importance of microenvironment-responsive acoustic sensitizers in amplifying antibacterial efficacy.

Furthermore, to overcome bacterial antioxidant defenses such as superoxide dismutase (SOD), Yang *et al.* engineered calcium carbonate-gallium-protoporphyrin IX (CaCO_3_-Ga-PPIX) nanospheres (CaGaPP NSs) incorporating polyethylene glycol (PEG). the infected microenvironment, CaGaPP NSs selectively released Ga^3+^ ions that functioned as "Trojan horse" agents to disrupt bacterial metabolism and inhibit SOD activity, thereby further enhancing the antibacterial activity of SDT 101. This work introduces a promising strategy that combines SDT nanomaterials with targeted suppression of bacterial systems for treatment of osteomyelitis.

In summary, SDT demonstrates significant antibacterial advantages through the combined effects of mechanical disruption mediated by US cavitation and ROS generation. Furthermore, owing to deep tissue penetration of US energy, SDT represents a particularly promising therapeutic strategy for osteomyelitis, especially for infections involving deep bone marrow lesions.

#### Challenges and prospects of SDT in osteomyelitis therapy

Although SDT strategy has gained increasing recognition for the treatment of osteomyelitis, several critical challenges remain to be addressed. Acoustic sensitizers represent a key determinant of SDT efficacy, yet current limitations related to complex preparation processes and unresolved *in vivo* biosafety concerns pose substantial barriers to clinical translation. Consequently, the rational design and development of multifunctional acoustic sensitizers remain central for future optimization of SDT-based osteomyelitis therapy. Emerging evidence indicates that MOF-based sonosensitizers integrated with hypoxia-adaptive SDT platforms offer promising solutions for refractory osteomyelitis. In particular, development of oxygen saturation-independent materials that leverage direct energy transfer or ROS catalytic amplification mechanisms has expanded the feasibility of SDT under hypoxic conditions. Future research should therefore prioritize systematic assessment of MOF biosafety and the development of degradable alternatives to support clinical translation. In addition, the existing US delivery systems lack the precision and operational flexibility required for consistent and convenient adjustment of acoustic parameters, limiting their suitability for clinical implementation. Development of next-generation US instruments with standardized and programmable acoustic parameters is thus another important direction for technological advancement. Moreover, the molecular mechanisms underlying SDT-induced apoptosis across different bacterial species remain incompletely understood and warrant further exploration.

In summary, SDT demonstrates considerable therapeutic promise for osteomyelitis owing to its superior tissue penetration ability. However, clinical efficacy is strongly influenced by the performance of US equipment and the stability of acoustic sensitizer delivery. In contrast, CDT, which does not rely on external energy input, enables sustained ROS generation and offers distinct advantages in the management of infections requiring continuous oxidative stress-mediated antimicrobial activity.

### CDT for osteomyelitis treatment

#### Overview of CDT in osteomyelitis treatment

In 2016, Shi *et al.* formally introduced the concept of CDT 102. Central to this therapeutic strategy are nanozymes, a class of nanoscale materials possessing intrinsic enzyme-like catalytic activity 103. Nanozymes integrate the physicochemical properties of nanomaterials with the catalytic functions of natural enzymes, enabling efficient catalysis of biological substrate reactions under mild physiological conditions. Compared with natural enzymes, nanozymes typically exhibit greater thermal and chemical stability, enhanced environmental tolerance, lower production costs, and favorable scalability for mass production 104. The discovery of nanozymes has challenged the traditional notion of inorganic materials as biologically inert and has revealed the inherent catalytic potential of nanomaterials, thereby expanding their utility across analytical chemistry, biosensing, environmental remediation, and biomedical applications. For example, cerium oxide (CeO_2_) and iron oxide (Fe_3_O_4_) nanoparticles with nanozyme activity have been widely explored for biosensing and therapeutic purposes 71. Based on material composition, nanoenzymes are broadly classified into metal-based nanoenzymes, carbon-based nanoenzymes, metal-MOF nanoenzymes, and bio-derived nanoenzymes 104. The core catalytic mechanisms underlying nanoenzyme-mediated CDT involve Fenton and Fenton-like reactions.

During the CDT process, nanocatalytic systems exploit the excess H^+^ or H_2_O_2_ present within pathological lesions to drive Fenton or Fenton-like reactions. In iron-based nanomaterials, Fe^2+^ ions are released and catalyze the decomposition of endogenous H_2_O_2_ into highly reactive ·OH under acidic conditions according to the reaction

Fe^2+^ + H_2_O_2_ → Fe^3+^ + ·OH + OH^-^

In addition to iron, other redox-active metal ions, such as Cu^+^, Mn^2+^, and Mo^6+^, can also catalyze H_2_O_2_ conversion into ·OH. As a highly cytotoxic Type I ROS, ·OH induces cellular damage through phospholipid peroxidation, mitochondrial dysfunction, and DNA damage, while simultaneously activating caspase-3-dependent apoptotic pathways, ultimately leading to programmed death of both target cells and pathogenic microorganisms. A large number of metal-based nanocatalytic systems has therefore been developed to enhance the efficiency of Fenton and Fenton-like reactions, thereby improving CDT therapeutic efficiency 102,105.

Initially established as an effective strategy for cancer therapy, CDT depends on acidic microenvironments and elevated H₂O₂ concentrations to sustain efficient ROS generation. Owing to the similar pathological features of tumor tissues and infected bone marrow lesions—characterized by mild acidity, hypoxia, and excessive H_2_O_2_ —CDT has demonstrated considerable promise for osteomyelitis treatment. Within osteomyelitis lesions, Fenton and Fenton-like catalysts convert endogenous H₂O₂ into ROS, thus exerting potent antibacterial and immunomodulatory effects. Compared with PDT and SDT, CDT-based nanomaterials enable continuous ROS generate without relying on external energy sources, providing a more durable and operationally straightforward therapeutic approach for osteomyelitis management.

#### Development of CDT nanotechnology for osteomyelitis treatment

##### CDT nanotechnology for osteomyelitis treatment via H₂O₂ supply

**Figure 5:**
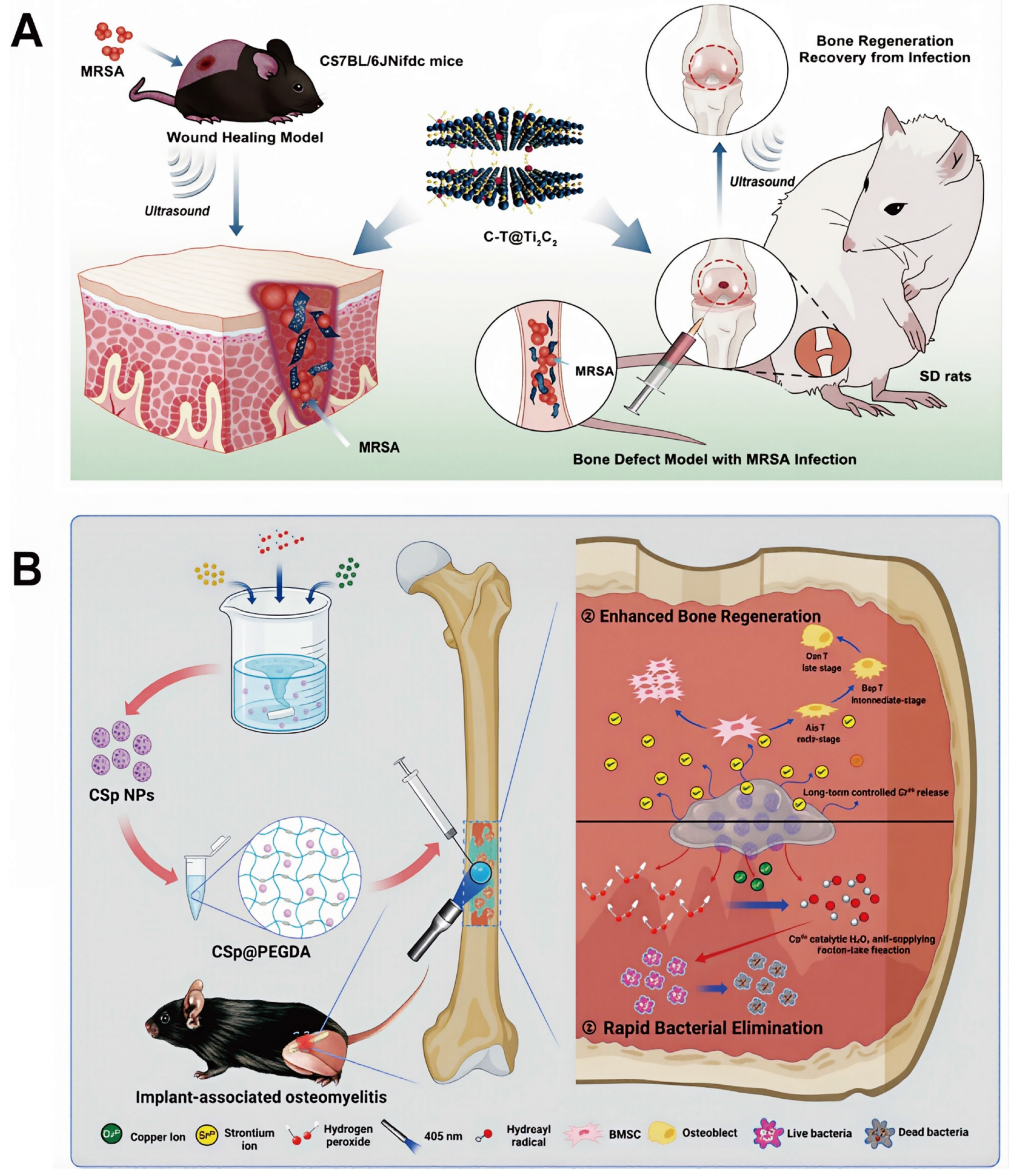
(A) Schematic illustration of the synthesis and therapeutic performance of C-T@Ti_3_C_2_ nanosheets, including *in vitro* and *in vivo* antibacterial activity and bone defect repair in deep infections. Adapted with permission from Ref. 106. Copyright 2023, Springer Nature. (B) Schematic illustration of the synthesis, antibacterial activity, and osteogenic performance of the CSp@PEGDA composite. Adapted with permission from Ref. 108. Copyright 2024, Wiley-VCH.

Supply of H₂O₂ at sites of infection improves the therapeutic efficacy of CDT. Numerous studies have therefore leveraged the intrinsic accumulation of H₂O₂ within osteomyelitis lesions to optimize CDT-based treatment strategies. For example, Yu *et al.* developed two-dimensional TiC nanosheets loaded with CaO_2_, which showed robust CDT activity through self-supplied H₂O₂ generation, resulting in effective antimicrobial action and substantial therapeutic benefit in osteomyelitis models 106 (**Figure 5A**).

##### CDT nanotechnology for osteomyelitis treatment dependent on acidic microenvironment

Efficient Fenton reactions require an acidic environment, typically with pH values ranging from 2 to 4, as elevated pH conditions inhibit nanomaterial decomposition and limit the release of catalytically active metal ions. The acidic microenvironment characteristic of osteomyelitis lesions therefore provides favorable conditions for high-efficiency CDT. For instance, Ge *et al.* constructed a multifunctional, pH-responsive drug delivery system by integrating zeolitic imidazolate framework-8 (ZIF-8) with celecoxib (CEL@ZIF-8). This platform enabled intelligent release of both metal ions and therapeutic agents in response to the acidic conditions at the infection site, ultimately achieving effective osteomyelitis treatment 107. Similarly, Guan *et al.* encapsulated copper-strontium peroxide nanoparticles (CSp) within PEG diacrylate (PEGDA) to generate CSp@PEGDA nanocomposites. These nanoparticles self-supplied H_2_O_2_ and released Cu²⁺ ions to initiate Fenton-like reactions, generating a large amount of ·OH for potent antibacterial activity. At the same time, the released Sr^2+^ ions enhanced osteogenic processes by promoting osteoblast proliferation, increasing alkaline phosphatase activity, facilitating extracellular matrix calcification, and upregulating gene expression related to bone formation 108 (**Figure 5B**).

Compared with tumor tissues, several intrinsic limitations restrict the application of CDT in osteomyelitis treatment, including relatively low endogenous H_2_O_2_ concentrations within bone infections and partial neutralization of the acidic microenvironmental by bone minerals and inflammatory responses. Accordingly, strategies involving autonomous H_2_O_2_ supplementation or glutathione depletion have proven effective in enhancing CDT efficiency. Furthermore, potential risks associated with metal accumulation and systemic toxicity arising from extensive use of transition metal catalysts, such as iron, copper, and manganese, warrant sustained investigation. Future development of CDT for osteomyelitis treatment should therefore focus on the design of highly efficient catalysts capable of operating under physiological or near-physiological conditions, the exploration of metal-free CDT nanozymes (e.g., carbon- and nitrogen-based structures), and the construction of intelligent nanoplatforms capable of dynamically sensing and modulating the pathological microenvironment. Integration of CDT strategies with osteogenic elements further represents a promising multifunctional therapeutic direction for osteomyelitis management.

#### Challenges and prospects of CDT in osteomyelitis treatment

Development of CDT for osteomyelitis therapy relies heavily on effective exploitation and modulation of the pathological microenvironment characterized by elevated H_2_O_2_ levels, increased GSH, and acidic pH. Although CDT has emerged as a promising therapeutic strategy osteomyelitis, several biosafety challenges must be addressed prior to clinical translation. First, the complex and heterogeneous microenvironment of osteomyelitis lesions may introduce unpredictable safety risks during CDT treatment. Second, insufficient targeting accuracy and limited specificity of current CDT nanomaterials increase the potential for off-target effects and damage to normal tissues. Third, extensive use of metal-based components in CDT nanomaterials raised concerns regarding metal accumulation and systemic toxicity *in vivo*. Consequently, future optimization of CDT for osteomyelitis should prioritize realistic modeling of lesion microenvironments, refinement of nanoscale targeting strategies, and development of non-metallic CDT nanomaterials with improved biosafety profiles.

In summary, the main advantage of CDT lies in its independence from external energy input; however, its antibacterial efficiency remains highly sensitive to local microenvironmental conditions, and reliance on a single therapeutic modality increase the risk of treatment failure. In contrast, MWDT offers the ability to deliver precise microwave-induced hyperthermia while simultaneously generating ROS for effective sterilization. Through the synergistic action of thermal effects and ROS-mediated antibacterial mechanisms, MWDT provides a more comprehensive and efficient therapeutic approach for the management of chronic osteomyelitis, combining deep tissue penetration with rapid therapeutic responses.

### MWDT for osteomyelitis treatment

#### Overview of MWDT in osteomyelitis treatment

Over the past decade, microwave activation has emerged as an increasingly attractive synergistic therapeutic modality due to its ability to induce both thermal effects and ROS, thereby enhancing antitumor and antimicrobial efficacy 109. In 2017, Fu *et al.* formally introduced the concept of MWDT, which integrates the hyperthermic advantages of conventional microwave thermal therapy (MTT) with ROS-mediated cytotoxic mechanisms, providing a more comprehensive and effective clinical strategy 110. ROS generation during MWDT is driven by both thermal and non-thermal effects associated with microwave irradiation. Microwave-induced heating accelerates ion migration and increases interionic collision frequency, leading to localized temperature rise and occurrence of redox reactions. Concurrently, microwave exposure alters cell membrane integrity and intracellular architecture, regulates the intracellular microenvironment, and further promotes ROS generation. Through these combined mechanisms, MWDT enables localized ROS generation at targeted tissue sites by simultaneously promoting redox reactions and interfering with intracellular signaling pathways 111,112.

In recent years, application of MWDT for the treatment of bacterial infections has expanded, with particularly encouraging outcomes reported in the treatment of osteomyelitis 113-119. Microwave irradiation offers substantial tissue penetration depth and effectively heats tissues with low electrical conductivity, high impedance, and low thermal conductivity, such as bone 120. Moreover, high microwave-to-thermal conversion efficiency enables rapid achievement of bactericidal temperatures exceeding 100 °C, thereby significantly shortening treatment duration 120. In parallel, extensive evidence confirms that microwave exposure promotes ROS generation 121. Collectively, these features establish microwave-responsive nanomaterials with unique physicochemical properties as promising platforms for advanced antimicrobial therapy of osteomyelitis.

#### Development of MWDT for osteomyelitis treatment

##### Design of MWDT nanomaterials

Microwave-absorbing materials convert microwave energy into thermal or other forms of energy through absorption and attenuation processes. The main mechanisms underlying microwave absorption include electromagnetic loss, magnetic loss, dielectric loss, and multiple reflection, which may operate individually or synergistically to confer efficient absorption performance 122,123. At present, microwave-responsive nanomaterials encompass both materials with intrinsic wave-absorbing properties and materials exhibiting microwave dynamic activity 124-126. Among these, nanomaterials with microwave dynamic properties are most frequently employed in the design and development of MWDT nanomaterials for osteomyelitis treatment. Nanomaterials with microwave dynamic properties usually possess H_2_O_2_-like enzymatic behavior or intrinsic microwave catalytic activity. For example, Mn-Zr-doped MOFs have been shown to act as H_2_O_2_-mimicking enzymes, catalyzing H_2_O_2_ decomposition into ·OH under microwave irradiation 164. This phenomenon is attributed to microwave-induced acceleration of electron transfer within the material, facilitating excitation of H_2_O_2_ molecules from the ground state and ultimately triggering rapid ·OH generation. In contrast, nanomaterials exhibiting microwave catalytic activity, such as Ga/In alloy nanostructures, are capable of directly producing ROS under microwave irradiation. This phenomenon arises from the formation of high-temperature “hot spots” on material surfaces created by localized resonant coupling of microwave energy, where electron transfer from Ga to H_2_O or O_2_ is promoted, thereby enhancing production of ·OH and superoxide anion radicals ·O_2_^-^ 109.

##### Application of MWDT nanomaterials in osteomyelitis treatment

**Figure 6:**
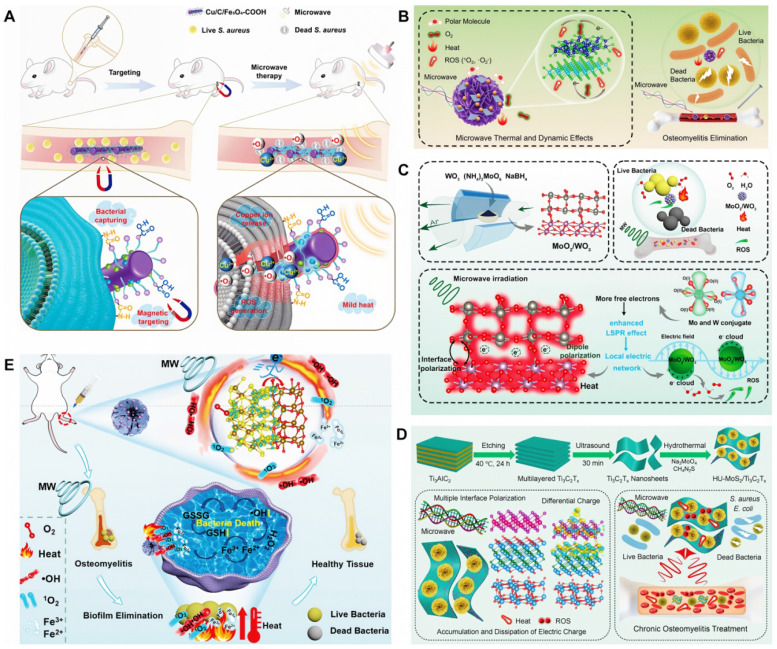
(A) Schematic diagram of Cu/C/Fe_3_O_4_-COOH for treatment of *S. aureus*-infected osteomyelitis. Adapted with permission from Ref. 127. Copyright 2024, Wiley-VCH. (B) Microwave-assisted therapeutic strategy for treating *S. aureus*-infected osteomyelitis using a microwave-responsive MoS_2_/FeS nanocomposite modified with rhein. Adapted with permission from Ref. 128. Copyright 2022, Wiley-VCH. (C) Schematic representation of MoO_2_/WO_3_ synthesis, microwave-mediated osteomyelitis treatment, and the combined mechanisms of microwave thermal and catalytic effects_._ Adapted with permission from Ref. 129. Copyright 2022, American Chemical Society. (D) Microwave-based treatment strategy for COM with *S. aureus* infection using MoS₂/Ti₃C₂Tₓ heterojunctions generated via hydrothermal treatment, featuring oxygen vacancy-rich TiO_2-x_ interfaces. Adapted with permission from Ref. 130. Copyright 2022, Wiley-VCH. (E) Schematic illustration of the preparation of Fe_2_O_3_/Fe_3_S_4_ composite materials. Adapted with permission from Ref. 131. Copyright 2024, Wiley-VCH.

MWDT nanomaterials used in osteomyelitis therapy are mainly categorized into magnetic loss materials and dielectric loss materials. Magnetic loss materials are typically represented by polymer composites filled with magnetic components such as ferrites or carbonyl iron powder. Notably, Fe_3_O_4_ nanoparticles have already received approval from the U.S. FDA for certain medical applications. Ren *et al.* developed an effective Cu/C/Fe_3_O_4_-COOH nanocomposite for osteomyelitis therapy, in which Fe_3_O_4_ nanoparticles with strong magnetic loss characteristics improved microwave absorption through optimized impedance matching of the dielectric matrix. Targeting of bacterial cells was achieved via interactions between surface -COOH groups on the nanocomposite and -NH_2_ groups on the bacterial walls. Under microwave irradiation, Cu/C/Fe_3_O_4_-COOH generated both thermal energy and ROS, resulting in disruption of bacterial membrane integrity and permeability. Antibacterial inhibition against *S. aureus* reached 99.99 ± 0.009% 127 (**Figure 6A**). Dielectric loss materials depend on tunable dielectric properties of carbonaceous or metallic fillers, which can be regulated through control of material thickness, depth, and filler composition. Jin *et al.* developed a microwave-responsive nanocomposite composed of MoS_2_/FeS and emodin (Rhein). Under microwave exposure, MoS_2_ promoted dipole polarization and ion conduction, resulting in molecular friction and dielectric loss, ultimately generating both thermal energy and ROS. The combined effects of singlet oxygen (^1^O_2_), superoxide anion (•O_2_^-^) and thermal energy achieved nearly 100% antibacterial efficacy against *S. aureus* and *E. coli* 128 (**Figure 6B**). Furthermore, binary metal oxides exhibit superior electrical conductivity and increased oxygen vacancy density compared with single metal oxides, thereby enhancing electron migration and providing abundant dipolar polarization sites. Based on the principle that interfacial polarization in binary metal oxides improves microwave absorption through enhanced conductivity loss, Zhu *et al.* designed a microwave-responsive MoO_2_/WO_3_ heterojunction for treatment of MRSA-induced osteomyelitis. Owing to tits high electrical conductivity, favorable dielectric loss characteristics, and excellent thermal stability, MoO_2_/WO_3_ achieved potent bactericidal activity and effective osteomyelitis treatment through the synergistic action of thermal effects and ROS generation. Inhibition rates against *S. aureus* and MRSA reached 99.27 ± 0.02% and 99.23 ± 0.43%, respectively 129 (**Figure 6C**).

In recent years, composite microwave-absorbing materials that integrate both magnetic and dielectric properties have been designed for osteomyelitis treatment to achieve synergistic antibacterial effects. For example, Jin *et al.* developed a MoS_2_/Fe_3_O_4_ composite to demonstrated effective therapeutic outcomes in osteomyelitis. The synergistic interaction between MoS_2_ and magnetic Fe_3_O_4_ facilitates multiple magnetic loss mechanisms, enhanced dielectric reflection, and interfacial polarization, thus conferring excellent microwave-induced thermal effects and efficient generation of ^1^O_2_ and ·O_2_^-^, which are essential for successful treatment of *S. aureus*-infected tibial osteomyelitis 130 (**Figure 6D**). Similarly, Liao *et al.* fabricated a Fe_2_O_3_/Fe_3_S_4_ composite material with strong microwave absorption capacity, enabling effective conversion of electromagnetic energy into heat. Upon microwave irradiation, differential charge accumulation and consumption at the Fe_2_O_3_/Fe_3_S_4_ interface enhance free electron release and subsequent binding with oxygen adsorbed on the composite surface, ultimately triggering a rapid burst of ROS. The combined magnetic loss and dielectric loss mechanisms promote simultaneous ROS generation and localized hyperthermia, leading to increased bacterial membrane permeability, oxidative stress induction, and eventual bacterial apoptosis. Antibacterial efficiencies of Fe_2_O_3_/Fe_3_S_4_ reached 99.87% against *S. aureus* and 99.59% MRSA 131 (**Figure 6E**).

By leveraging the deep tissue penetration properties of microwave radiation, MWDT enables simultaneous induction of localized hyperthermia and ROS-mediated antibacterial effects, generating a synergistic therapeutic outcome that substantially exceeds the efficacy of either modality alone. The high energy of microwave irradiation overcomes the penetration limitations inherent to phototherapy and demonstrates substantial potential for treating deep-seated and refractory bone infections.

#### Challenges and prospects of MWDT in osteomyelitis treatment

Several key challenges currently limit the clinical translation of MWDT for osteomyelitis therapy. First, existing MWDT platforms lack precise targeting capability toward infected lesions. Second, currently available microwave sensitizers often exhibit insufficient microwave responsiveness and suboptimal thermal conversion efficiency, thereby limiting therapeutic effectiveness in osteomyelitis management. Third, inadequate control of heat distribution during microwave irradiation introduces the risk of collateral thermal injury to surrounding healthy tissues or vital organs. Consequently, incorporation of advanced imaging probes and targeting moieties is expected to facilitate the development of multifunctional micro-and nanomaterials, enabling more precise, efficient, and safe MWDT techniques for osteomyelitis therapy.

In summary, the four major ROS-based therapeutic strategies discussed above—PDT, SDT, CDT, and MWDT—each exhibit unique advantages while also presenting unavoidable limitations. Future osteomyelitis treatment paradigms are therefore likely to evolve toward multimodal combination therapies to overcome the shortcomings associated with single-modality interventions. Integration of PDT, SDT, CDT, and MWDT provides expanded opportunities for personalized osteomyelitis treatment, with particular promise for addressing chronic, deep-seated, and refractory infections. Continues technological innovation and development of advanced therapeutic modalities are expected to further refine osteomyelitis treatment strategies, ultimately improving clinical outcomes and overall patient quality of life.

### Combination of different ROS nanotechnologies for osteomyelitis treatment

Although ROS-based nanotechnologies, including PDT, SDT, CDT, and MWDT, have shown substantial therapeutic potential and promising application prospects in osteomyelitis management, single-modality dynamic treatment strategies remain insufficient to achieve rapid and comprehensive control of complex microbial infections. Consequently, contemporary ROS-based therapeutic approaches for osteomyelitis have gradually shifted from monotherapy toward multimodal combination strategies to enhance infection eradication and promote effective wound healing. Integration of complementary dynamic treatment strategies enables mutual compensation for the inherent limitations of individual approaches and facilitates synergistic interactions among different ROS-generating mechanisms and auxiliary therapeutic components, thereby producing a therapeutic outcome that exceeds the efficacy of any single strategy alone. This section summarizes current combinatorial strategies involving multiple ROS-based nanotechnologies, with the aim of elucidating their synergistic mechanisms and evaluating the potential of integrated therapeutic platforms in addressing the multifaceted challenges posed by osteomyelitis-associated infections.

#### Combination of PDT and SDT for osteomyelitis treatment

SDT offers superior tissue penetration compared with PDT, whereas PDT benefits from photosensitizers that exhibit hinger intrinsic ROS generation efficiency 132,133. Consequently, combination of PDT and SDT enables simultaneous optimization of tissue penetration and ROS generation, making this combination particularly suitable for the treatment of osteomyelitis with deep tissue involvement. Ding *et al.* synthesized barium titanate nanotubes (BNTs) through a combined anodic oxidation and hydrothermal process, achieving efficient ROS generation and potent antibacterial activity under both US and NIR irradiation via synergistic piezoelectric and pyroelectric effects. The coordinated action of PDT and SDT further enhanced charge transfer and electron-hole pair separation, thereby amplifying the coupled pyroelectric-piezoelectric catalytic effect and markedly increasing ROS output. The resulting ROS burst induces extensive structural and functional damage to bacterial DNA and proteins, eventually leading to irreversible bacterial cell death. *In vivo* experiments showed that the antibacterial efficacy of BNTs reached up to 99% 134.

#### Combination of SDT and CDT for osteomyelitis treatment

**Figure 7:**
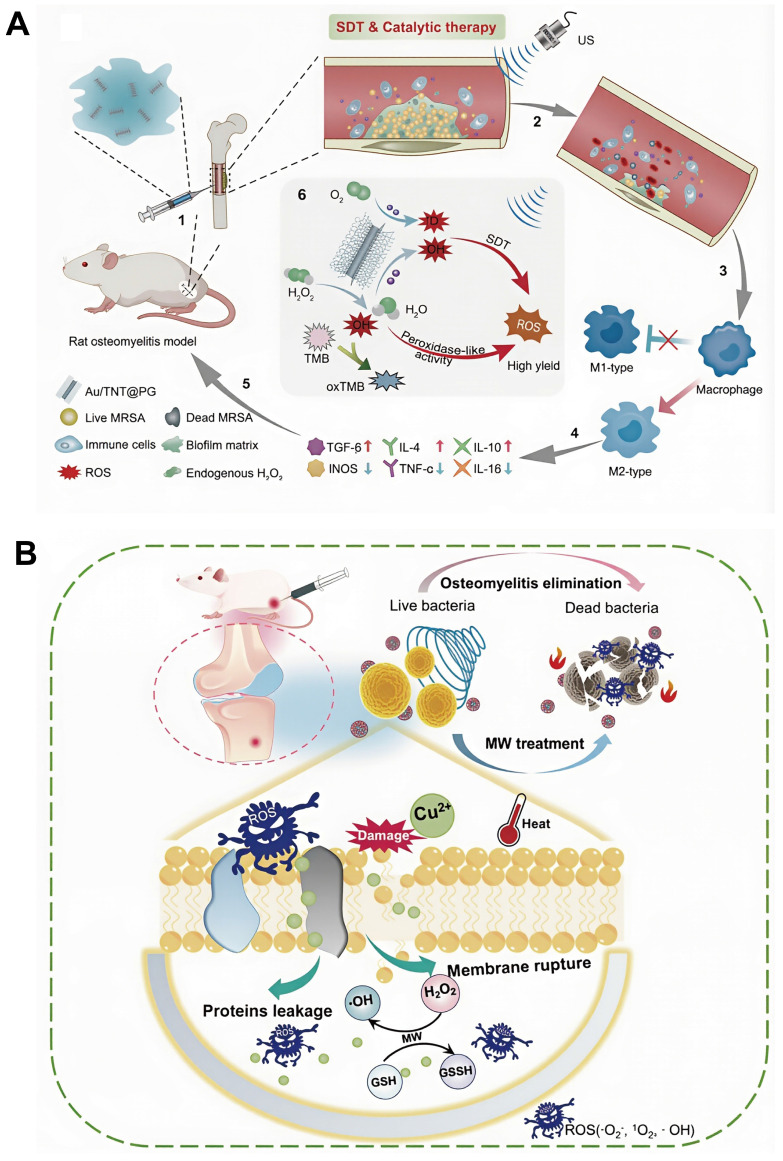
** (**A) Construction and therapeutic mechanism of Au/TNT@PG nanostructures for efficient osteomyelitis treatment. Adapted with permission from Ref. 135. Copyright 2022, Wiley-VCH. (B) Development of microwave-responsive CuCeOₓ materials for the treatment of *S.aureus*-infected osteomyelitis. Adapted with permission from Ref. 137. Copyright 2023, Wiley-VCH.

Previous studies have reported that localized turbulence generated by US shock waves can significantly improve the efficiency of Fenton reactions. The observation has provided a strong foundation for the development of synergistic strategies integrating SDT with CDT to achieve antimicrobial outcomes. For example, Cheng *et al.*, conjugated a guanidin-rich polymer (PG) onto gold-doped titanate nanotubes (Au/TNTs) capable of absorbing US, thereby constructing multifunctional nanostructures (Au/TNT@PG) that simultaneously exhibit sonodynamic properties and peroxidase-like catalytic activity. US absorption by Au/TNT@PG markedly enhanced CDT efficiency and ·OH generation. In addition, the biofilm-penetrating characteristics of PG facilitated efficient biofilm disruption and induction of bacterial apoptosis, resulting in superior antibacterial efficacy 135 (**Figure 7A**).

#### Combination of MWDT and CDT for osteomyelitis treatment

Microwave-induced hypothermia has been shown to effectively destroy bacterial biofilm structure as well asl cell wall and membrane integrity, thereby improving the antibacterial efficacy of ROS. Building upon this principle, Wei *et al.* developed a sodium (Na^+^)-rich Prussian blue (PB) nanomaterial that functions as both a microwave-absorbing agent and an iron ionophore for the treatment of bacterial osteomyelitis. PB nanoparticles absorb microwave energy through dielectric loss and mesoporous structural reflection, generating heat that increases bacterial membrane permeability and accelerates intracellular uptake of iron ions. This process subsequently enhances Fenton reaction efficiency and ROS generation, thus leading to bacterial apoptosis. Antibacterial inhibition rates of PB nanoparticles reached 99.08% against *S. aureus* and 99.67% against *E. coli* 136. In addition, cerium-based compounds, owing to their favorable dielectric polarization, dielectric relaxation, and conductive loss properties, have been identified as promising microwave-responsive antibacterial materials. Sun *et al.* designed a CuCeOₓ composite oxide for the treatment of *S. aureus*-induced osteomyelitis. Under microwave irradiation, CuCeOₓ simultaneously produced thermal energy and ROS, thereby increasing Cu^2+^ thermal sensitivity, enhancing bacterial membrane permeability, and amplifying Fenton-like reaction efficiency, resulting in substantial accumulation of ·OH within bacterial cells. The combined MWDT-CDT treatment achieved an antibacterial efficacy of 99.98% ± 0.02% against *S. aureus* 137 (**Figure 7B**).

In summary, investigation of synergistic ROS-based therapeutic strategies, including PDT-SDT, SDT-CDT, and MWDT-CDT combinations, holds significant promise for achieving breakthroughs in the treatment of deep-seated and refractory osteomyelitis.

This review provides a comprehensive analysis examines multimodal ROS-based therapeutic strategies, thereby complementing existing literature on ROS therapies for osteomyelitis, which has largely emphasized single-modality approaches and consequently offered limited insight into enhancing bone infection permeability and microenvironmental modulation. The present work not only elucidates the synergistic mechanisms and representative case studies of diverse ROS-based therapeutic modalities for osteomyelitis management but also critically examines the complementary strengths and translational challenges inherent to each ROS technology. Collectively, these perspectives establish multimodal ROS therapy as a key developmental direction for next-generation osteomyelitis treatment strategies.

### ROS-based diagnostic strategies for osteomyelitis

In recent years, a variety of ROS-responsive diagnostic approaches has been explored, primarily at the preclinical stage, aiming to improve early detection, lesion localization, and therapeutic monitoring of osteomyelitis 138. Based on detection principles and imaging modalities, current ROS-based diagnostic tools are broadly categorized into ROS-responsive optical imaging probes and ROS-activated MRI contrast agents.

#### ROS-responsive optical imaging strategies

ROS-responsive optical probes constitute the most extensively studied class of ROS-based diagnostic tools. These probes are typically designed with ROS-sensitive chemical moieties, such as boronate esters, thioketal linkers, and sulfur-containing functional groups, which undergo structural cleavage or oxidation in the presence of elevated ROS levels, thereby generating fluorescence or photoacoustic signals. Although ROS-responsive fluorescent probes have not yet been widely validated in dedicated osteomyelitis models, substantial progress achieved in infection-related and orthopedic infection imaging provides important references and strong translational potential for their future application in osteomyelitis diagnosis and therapeutic monitoring.

Currently, a representative class of ROS-responsive fluorescent probes consists of ROS-activatable NIR fluorescent nanoprobes, which remain optically quenched during systemic circulation but become fluorescent upon oxidative activation at sites of infection. Bouché et al. reported a hybrid nanogel formulation incorporating gold nanoparticles (AuNPs), in which ROS-triggered disintegration or cross-linking of the nanogels enabled quantitative diagnostic detection through corresponding changes in signal intensity 139. Xu et al. developed fluorescence-quenched TK-CBT nanoparticles containing a thioketal trigger moiety 140. Elevated ROS levels within S. aureus-infected macrophages induced nanoparticle fragmentation and activated NIR fluorescence, thereby enabling noninvasive *in vivo* imaging of S. aureus infection. This "ROS-triggered dequenching" paradigm supports the feasibility of exploiting localized oxidative stress as an endogenous activation signal for infection-specific optical diagnosis, which is highly relevant to S. aureus-dominant osteomyelitis. Beyond ROS-activatable probes, studies in orthopedic infection models have also highlighted the utility of NIR fluorescence tracers for precise localization and image-guided surgical debridement, providing valuable reference for osteomyelitis management. Park et al. compared two fluorescent probes targeting staphylococcal biofilm-associated implant infections, including a labeled antibiotic (Vanco-800CW) and an antibody-based tracer (1D9-680) directed against a staphylococcal antigen, and demonstrated their feasibility for fluorescence-guided debridement 141. Integration of such NIR fluorescence tracers with ROS-responsive probes is anticipated to further enhance the precision of osteomyelitis localization and diagnostic accuracy in future clinical applications.

At present, most ROS-responsive fluorescent probes investigated for osteomyelitis remain at the preclinical stage, primarily constrained by several limitations: (i) limited tissue penetration and optical attenuation in deep bone lesions; (ii) the risks of false-positive signals arising from elevated ROS levels associated with sterile inflammation or post-surgical tissue damage; and (iii) challenges related to batch-to-batch reproducibility and long-term biosafety of nanoprobes. Future developments of ROS-responsive fluorescent probes for osteomyelitis diagnosis are therefore expected to emphasize the use of NIR-I/NIR-II fluorophores, implementation of dual-locked activation designs that combine bacteria targeting with ROS responsiveness, and incorporation of multimodal imaging readouts, such as fluorescence integrated with photoacoustic imaging or MRI, to improve diagnostic robustness under the complex pathological conditions characteristic of osteomyelitis.

#### ROS-activating MRI contrast agents

Current ROS-activating MRI strategies mainly rely on ROS-triggered release of paramagnetic metal ions and the consequent enhancement of T1-weighted signals. Among reported platforms, MnO2-based nanostructures are considered particularly promising and have been successfully applied in osteomyelitis models. These ROS-activatable MRI contrast agents utilize the elevated H2O2 levels and acidic microenvironment characteristic of infected bone tissue to promote redox-mediated decomposition of MnO2 and generation of paramagnetic Mn2+ ions, thereby significantly enhancing T1-weighted MRI contrast.

Beyond bone-related applications, a broader range of ROS- or enzyme-activatable MRI contrast agents has been developed beyond bone-related models. Mn-based ROS-responsive probes, including Mn-TyEDTA derivatives, showed pronounced increases in longitudinal relaxivity following peroxidase-mediated radical polymerization in the presence of H2O2 142. Compared with normal tissues, these probes exhibit selective MRI signal amplification within inflamed regions, supporting the feasibility of synergistic MRI activation through combined enzymatic and ROS-mediated mechanisms.

Accordingly, detection of abnormal ROS levels may serve as a valuable diagnostic indicator for chronic osteomyelitis, enhancing the clinical utility of conventional inflammatory biomarkers. Advances in nanotechnology and development of targeted molecular probes have introduced new possibilities for ROS-driven, disease-specific diagnosis. Nevertheless, challenges related to biosafety, stability, and standardization have confined most ROS-based diagnostic nanoprobes for osteomyelitis to the preclinical stage. Moreover, the nonspecific clinical presentation of atypical osteomyelitis and the limited specificity of ROS-associated signals necessitate confirmatory verification through imaging and histopathological examination during diagnostic decision-making. Integration of ROS-specific molecular imaging approaches, such as responsive nanoprobes, with metabolomic profiling of mitochondrial function and AI-assisted data analysis is expected to substantially enhance the sensitivity of early osteomyelitis detection. Although current ROS-based diagnostic strategies continue to face challenges in specificity and translational feasibility, the central role of ROS in osteomyelitis pathophysiology, combined with emerging technologies and multidimensional data integration, offers strong potential to overcome existing diagnostic bottlenecks and advance the development of personalized treatment strategies.

### Combination of ROS nanotechnology with antibiotics for osteomyelitis treatment

As a refractory infection of bone tissue, osteomyelitis presents multiple challenges to the development of effective therapeutic strategies, including limited antibiotic penetration due to bacterial biofilm formation, continuous emergence of drug-resistant strains, and systemic toxicity associated with conventional systemic antibiotic administration. Despite these limitations, antibiotics remain the cornerstone of osteomyelitis management because of their established efficacy and favorable cost profile. However, excessive and prolonged antibiotic use has contributed to the occurrence of chronic osteomyelitis and treatment failure in other infectious diseases, highlighting the urgent need for alternative or adjunctive therapeutic approaches. In this context, ROS-based nanotechnology offers a promising avenue for improving antimicrobial efficacy and overcoming antibiotic resistance. Therefore, increasing attention has been directed toward the development of synergistic treatment strategies that integrate ROS nanotechnology with antibiotic therapy for osteomyelitis. This section systematically discusses the molecular mechanisms underlying microbial antibiotic resistance, recent advances in nanotechnology for reversing antibiotic resistance, and emerging strategies that combine ROS nanotechnology with conventional antibiotics to enhance therapeutic outcomes in osteomyelitis management.

#### Mechanism of microbial resistance to antibiotics

The emergence and global dissemination of antibiotic-resistant microorganisms represent a critical threat to human health and have evolved into a major public health crisis that requires coordinated international response. Antibiotic resistance compromises the effectiveness of conventional antimicrobial therapies against common pathogens. For instance, infections caused by drug-resistant *Mycobacterium tuberculosis* necessitate prolonged multidrug treatment regimens, often accompanied by severe side effects 143. In addition, antibiotic-resistant infections are associated with extended hospitalization, significantly increased healthcare costs, and elevated mortality risks. Notably, MRSA frequently exhibits cross-resistance to multiple antibiotic classes, further complicating treatment and markedly increasing patient mortality 144.

Bacterial antibiotic resistance arises through multiple mechanisms and is generally classified into intrinsic resistance, acquired resistance, and adaptive resistance. Intrinsic resistance refers to the natural insensitivity of certain bacterial species to specific antibiotics, typically due to inherent structural or metabolic characteristics, such as absence of appropriate drug targets. Acquired resistance develops when previously susceptible bacteria undergo genetic mutations or acquire resistance genes through horizontal gene transfer. Adaptive resistance, in contrast, is a transient phenotype induced by environmental stimuli, including antibiotic exposure, growth phase, pH fluctuations, ionic conditions, and nutrient availability. Unlike intrinsic and acquired resistance, adaptive resistance reflects a reversible stress response that diminishes once the inducing environmental pressure is removed. The underlying mechanisms driving microbial antibiotic resistance primarily involve reduction of intracellular antibiotic concentrations, modification of antibiotic targets, enzymatic inactivation or structural modification of antibiotics, and activation of alternative metabolic pathways that bypass antibiotic targets 145-147.

##### Decrease of intracellular antibiotic concentration

Effective antibacterial activity requires antibiotics to penetrate bacterial cells and reach their intracellular targets. Reduced membrane permeability and enhanced activity of efflux pumps can markedly lower intracellular antibiotic concentrations, thereby promoting the development of bacterial resistance 148,149. These processes constitute key physicochemical defense strategies that increase bacterial survival by restricting intracellular drug accumulation. Elucidation of these resistance mechanisms provides critical theoretical foundation for the rational design of next-generation antimicrobial agents and strategies aimed at reversing antibiotic resistance, with significant implications for global public health.

**Decreased antibiotic permeability**: Antibiotic permeability refers to the ability of antimicrobial agents to traverse bacterial cell envelopes and access intracellular targets. Reduced permeability represents an adaptive survival strategy that bacteria developed under antibiotic selective pressure. Decreased permeability of the cell wall or membrane limits antibiotic penetration, thus significantly lowering intracellular drug concentration and diminishing bactericidal efficacy. In Gram-positive bacteria, the cell envelope comprises a thick peptidoglycan layer and a single cytoplasmic membrane. In contrast, Gram-negative bacteria possess a more complex barrier structure consisting of an inner membrane and an additional outer membrane, which confers increased resistance to antimicrobial entry and contributes to the generally lower antibiotic susceptibility observed in Gram-negative bacteria. Porins constitute the primary transport channels that facilitate diffusion of hydrophilic antibiotics, such as β-lactam, fluoroquinolones, tetracyclines, and chloramphenicol, across the outer membrane of Gram-negative bacteria. Consequently, porin loss or decreased porin expression represents a major mechanism of antibiotic resistance. Under physiological conditions, outer membrane proteins OmpF and OmpC in *E. coli* form nonspecific transmembrane channels that permit entry of antibiotics and other small molecules 150. However, repeated antibiotic exposure increases the likelihood of mutations in genes encoding OmpF, resulting in reduced channel expression or complete loss of function, thereby conferring resistance to antibiotics such as β-lactams and quinolones 151. Accordingly, regulation of porin expression represents a key target for the development of future strategies to combat antibiotic resistance.

**Promotion of antibiotic efflux**: Antibiotic efflux refers to the active expulsion of intracellular antimicrobial agents via efflux pumps. These pumps are transmembrane transport proteins that utilize proton gradient or ATP hydrolysis to export antibiotics either selectively or broadly. Many efflux systems are capable of expelling structurally diverse antibiotics, thereby conferring multidrug resistance. In Gram-negative bacteria, efflux pumps operate in concert with the dual-membrane envelope to produce particularly robust antibiotic resistance phenotypes. Five major transporter families are implicated in antibiotic efflux: resistance-nodulation-division (RND), major facilitator super family (MFS), small multidrug resistance (SMR), multidrug and toxic compound extrusion (MATE), and ATP-binding cassette (ABC) transporters 152. Alterations in porin expression alone typically induce only low-level resistance; however, reduced antibiotic uptake due to porin loss markedly amplifies the effectiveness of coexisting resistance mechanisms, including efflux pump activity and enzymatic antibiotic degradation, thereby promoting high-level resistance 153. In addition, efflux pumps exhibit substrate specificity. Consequently, effective reversal of certain antibiotic resistance phenotypes development of therapeutic strategies that specifically target relevant efflux systems, which represent important molecular targets for overcoming antimicrobial resistance 154.

##### Inactivation or modification of antibiotics

Another common mechanism of bacterial antibiotic resistance involves enzymatic inactivation or structural modification of antimicrobial agents, leading to loss of drug efficacy. β-Lactamases represent a classical example of this mechanism, hydrolyzing the β-lactam ring of β-lactam antibiotics and thereby disrupting the amide bond essential for antimicrobial activity. In addition to β-lactamases, a variety of bacterial enzymes catalyze chemical modifications of antibiotics through group transfer reactions, ultimately conferring resistance. Rifampicin resistance provides a well-characterized illustration of this process. Rifampicin can be rendered inactive through modification by ADP-ribosyltransferases, glycosyltransferase, phosphotransferase, and monooxygenase 155. In *M. abscess*, ADP-ribosyltransferase catalyzes ribosylation at the C23 hydroxyl group of rifampicin, obstructing its binding to RNA polymerase, thus promoting rifampicin resistance 156. Glycosyltransferases can similarly modify the C23 hydroxyl group, producing comparable resistance effects 157. Furthermore, phosphotransferases convert the C21 position of rifampicin into an inactive form, while monooxygenases diminish antibacterial efficacy by oxygenating the naphthol moiety of rifampicin and preventing its binding to the RpoB subunit of RNA polymerase 158.

##### Modification of antibiotic targets

High-affinity interaction between antibiotics and their molecular targets is fundamental to antimicrobial efficacy. Genetic mutations or biochemical modifications of antibiotic targets can substantially reduce drug-binding affinity, representing a major mechanism underlying antibiotic resistance. For example, methicillin-resistant *S. aureus* (MRSA) acquires resistance to β-lactam antibiotics through production of an altered penicillin-binding protein (PBP2a) with markedly reduced affinity for β-lactams 159. Similarly, ribosomal target modification contributes significantly to antibiotic resistance. In *E. coli*, ribosomal methyltransferases catalyze mono- or dimethylation of the A2058 residue within 23S rRNA of the large ribosomal subunit, thereby obstructing antibiotic binding and conferring resistance 160. In addition, increased methylation of 16S rRNA mediated by ribosomal methyltransferases reduces bacterial susceptibility to macrolide antibiotics 161 and is also strongly associated with high-level resistance to aminoglycosides 162.

##### Evasion of metabolic pathways targeted by antibiotics

To mitigate the inhibitory effects of antibiotics essential metabolic pathways, bacteria employ a series of adaptive strategies, including acquisition of alternative genes that bypass antibiotic targets. Methicillin, for instance, exerts antibacterial activity by binding to penicillin-binding proteins (PBPs) and inhibiting their transeptidase activity, thereby disrupting bacterial cell wall synthesis. However, MRSA overexpresses PBP2a, a homologous variant of PBP that exhibits markedly reduced affinity for methicillin. Binding of methicillin to PBP2a fails to inhibit transpeptidase activity, allowing cell wall synthesis to proceed and conferring high-level resistance.

#### Nanotechnology for reversing antibiotic resistance

Nanotechnology-based strategies to overcome antibiotic resistance mainly focus on the development of nanoparticles with intrinsic antibacterial ability to either replace conventional antibiotics or potentiate the efficacy of existing antimicrobial agents. In 2011, Huh *et al.* introduced the concept of “nanoantibiotics”, referring to nanomaterials that exhibit direct antibacterial activity or function as novel antibiotic platforms, showing potent bactericidal effects against drug-resistant pathogens with minimal propensity for resistance development 163. Nanoantibiotics mitigate antibiotic resistance through multiple mechanisms, including enhancement of intracellular antibiotic uptake, reduction of bacterial efflux, disruption of the biofilm structures, and precise targeting of infection sites 164. In addition, nanotechnology-based delivery systems reduce the likelihood of resistance development by enabling localized and controlled antibiotic release at infected tissues, while simultaneously improving the stability, solubility, and biocompatibility of pharmacologically challenging antibiotics, thereby enhancing their overall therapeutic efficacy.

##### Nanotechnology for promotion of antibiotic penetration or reduction of antibiotic efflux

Nanocarriers such as liposomes and dendrimers can simultaneously promote antibiotic penetration across bacterial membranes and suppress antibiotic efflux. Liposomes, which are spherical vesicles composed of one or more lipid bilayers, facilitate antibiotic entry into microbial cells through rapid fusion with bacterial membranes. This mechanism enables accelerated intracellular delivery and achievement of high local antibiotic concentrations, which can saturate and overcome transmembrane efflux pumps responsible for antibiotic extrusion. Consequently, liposomal delivery systems effectively counteract efflux-mediated resistance mechanisms. Dendritic polymers, characterized by highly branched architectures and large specific surface areas, represent another class of efficient antibiotic delivery vehicles. Quaternary ammonium-functionalized dendrimers, bearing abundant surface-positive charges, readily interact with the negatively charged bacterial cell envelope, thereby increasing membrane permeability and promoting intracellular antibiotic accumulation 164.

##### Nanotechnology for destroying biofilms

Biofilms are highly organized bacterial communities encapsulated within an extracellular polymeric substance (EPS) matrix composed of polysaccharides, proteins, and extracellular DNA. The dense EPS barrier severely restricts antibiotic penetration, allowing conventional therapies to eliminate primary surface-layer bacteria while leaving deeply embedded microbial populations largely unaffected. This limited penetration not only compromises therapeutic efficacy but also accelerates bacterial evolution and emergence of antibiotic resistance. Consequently, effective management of biofilm-associated bacterial infections requires antimicrobial strategies that surpass the capabilities of conventional antibiotics. Recent advances in nanotechnology have demonstrated substantial anti-biofilm potential. Abdelghafar *et al.* reported that ZnO nanoparticles significantly suppressed biofilm formation by *S. aureus*, with marked down regulation of key biofilm-related genes, including *icaA*, *sarA*, *katA*, and *sigB* 165. Xiu *et al.* developed an US-responsive catalytic microbubble system composed of a piperacillin-containing shell incorporating Fe_3_O_4_ nanoparticles surrounding an air core for the treatment of chronic *P. aeruginosa* biofilm-associated lung infections. Under US stimulation, these micro bubbles disrupted biofilm architecture and enhanced penetration of both Fe_3_O_4_ nanoparticles and piperacillin into deep biofilm layers. Concurrently, Fe_3_O_4_ nanoparticles catalyzed H_2_O_2_ decomposition to produce free radicals, further degrading the biofilm matrix and synergistically enhancing antibacterial activity 166. Collectively, these studies highlight the unique advantages of nanomaterials in promoting biofilm penetration, matrix degradation, and regulatory modulation of biofilm-related gene expression. Moreover, they support the development of highly targeted, multimodal antibacterial systems that integrate molecular intervention with physical biofilm disruption, offering promising new strategies for combating biofilm-associated infections.

##### Nanotechnology targeting the infection site

Infection-targeted nanotechnology enables precise drug delivery to infection sites, thereby enhancing local antimicrobial efficacy while reducing systemic exposure and the likelihood of resistance development. Bacterial targeting strategies commonly exploit physicochemical charge interactions, glycosylated motifs, specific surface antigens, corresponding antibodies, or signature enzymes, and have been successfully evaluated across numerous experimental and clinical investigations 167-170. For example, Hussain *et al.* adopted phage display technology to screen out a cyclic nine-amino acid peptide (CARG), with high specificity for *S. aureus*, facilitating development of targeted antibiotic delivery platforms with enhanced antibacterial effects. CARG-functionalized porous silicon nanoparticles loaded with vancomycin preferentially accumulated in *S. aureus*-infected lung and skin tissues compared with uninfected tissues or *Pseudomonas aeruginosa*-infected tissues 171. This approach significantly reduced required systemic antibiotic doses and associated side effects while improving therapeutic efficacy against multidrug-resistant bacteria, including MRSA. Similarly, Jayawardena *et al.* enhanced nanoparticle binding and internalization in *E. coli* by conjugating maltotriose to various nanomaterials, including SiO_2_ nanoparticles, FeOₓ nanoparticles, and SiO_2_-coated quantum dots, achieving broad applicability across multiple strains of *E. coli* 172. Collectively, infection-targeted nanotechnologies have transformed antimicrobial treatment paradigm through precision delivery strategies that improve eradication of multidrug-resistant pathogens while simultaneously reducing antibiotic selection pressure and resistance evolution, providing an innovative and highly promising solution to the escalating antimicrobial resistance crisis.

#### ROS nanotechnology for combined antibiotic treatment of osteomyelitis

**Figure 8:**
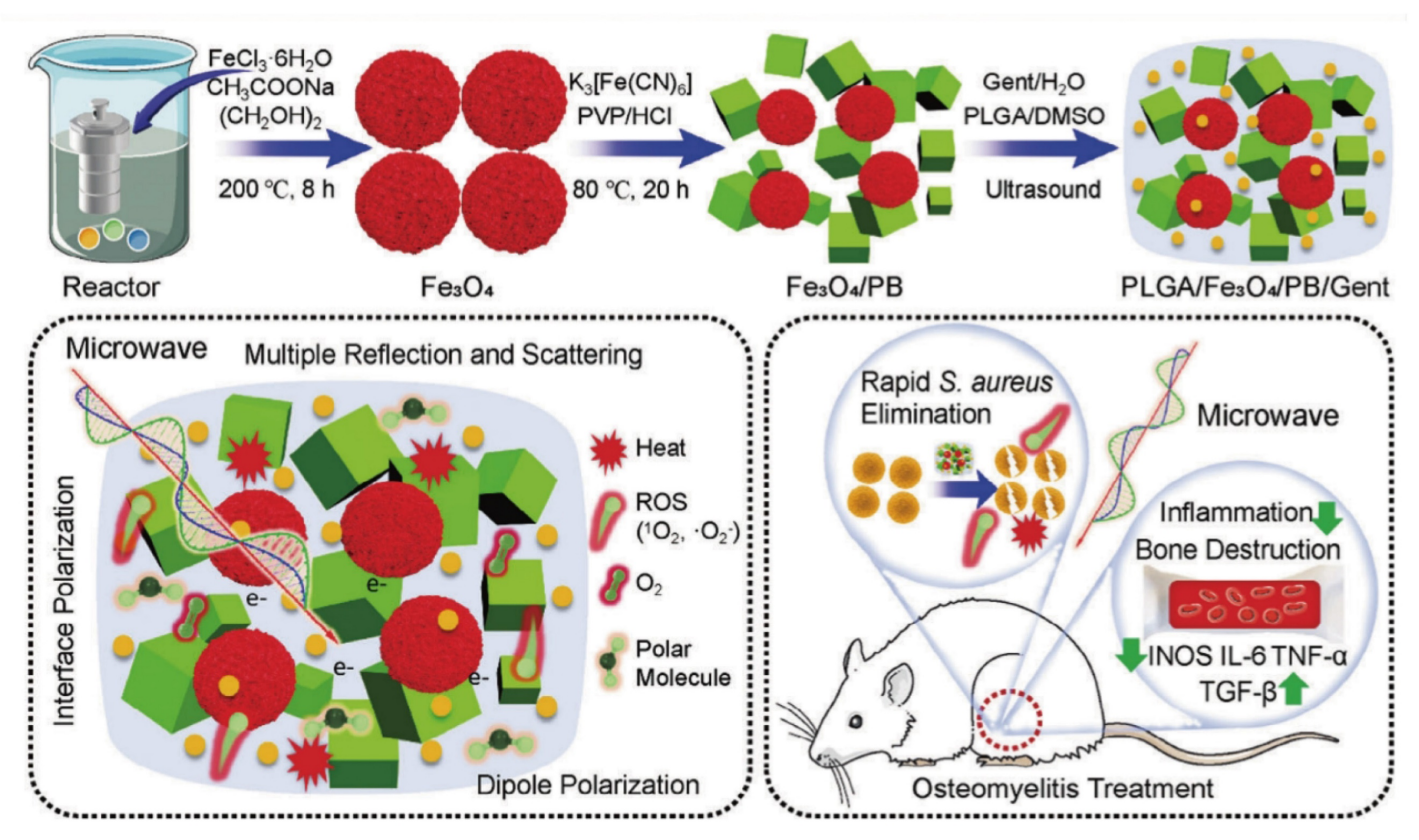
Schematic diagram of the microwave-responsive, magnetically targeted composite PLGA/Fe3O4/PB/Gent integrating MTT, MDT, and CT for eradication of acute *S aureus*-infected osteomyelitis. Adapted with permission from Ref. 173. Copyright 2024, Wiley-VCH.

Current antibiotic-based treatment strategies for osteomyelitis continue to face substantial clinical limitations. Destruction of cortical bone vasculature often leads to insufficient drug accumulation at infection sites following systemic administration, creating so-called "drug desert” regions. In addition, bacterial biofilm severely restricts antibiotic penetration. Prolonged high-dose antibiotic therapy further contributes to antimicrobial resistance, hepatotoxicity, nephrotoxicity, and other side effects. As discussed earlier, antibiotic resistance may be mitigated through increased intracellular antibiotic uptake, suppression of drug efflux, biofilm disruption, and precise targeting of infection sites. ROS-based nanotechnology offers strong potential to reverse antibiotic resistance due to its capacity for membrane disruption and broad-spectrum antimicrobial action. When combined with targeted ligands or antibiotics, ROS nanoplatforms further demonstrate enhanced therapeutic precision and efficacy. Sustained-release nanomaterials have been widely used in clinical management of chronic osteomyelitis owing to their ability to maintain prolonged minimal inhibitory concentrations at infection sites while minimizing off-target toxicity. For example, Lu *et al.* synthesized a multifunctional nanoagent composed of bovine serum albumin-manganese dioxide-ubiquicidin29-41-indocyanine green-gentamicin (BMUIG). The antimicrobial peptide ubiquicidin29-41 selectively binds bacterial surfaces, directing the nanocomplex to infected tissues, where abundant ROS generation combined with low-dose gentamicin achieved effective osteomyelitis treatment 87. Similarly, Jin *et al.* reported a microwave-responsive magnetic targeting composite system composed of Fe_3_O_4_/PB nanoparticles, gentamicin, and biodegradable poly(lactic-co-glycolic acid) (PLGA). Under microwave irradiation, synergistic thermal and ROS effects generated by Fe_3_O_4_/PB nanoparticles, together with magnetically guided release of gentamicin, produced potent antibacterial activity against osteomyelitis 173 (**Figure 8**). Collectively, ROS-responsive drug delivery systems offer dual therapeutic advantages for osteomyelitis management: precise lesion targeting that markedly enhances local drug concentration and sustained antibacterial activity mediated by controlled release, intrinsic bioactivity, and microenvironmental responsiveness. This "precision strike" treatment paradigm represent a fundamental breakthrough beyond conventional systemic antibiotic therapy. Future studies should prioritize elucidation of dynamic nanocarrier release mechanisms within complex infection microenvironments, comprehensive long-term biosafety evaluation, and optimization of clinical translation pathways to enable progression from "passive drug loading" toward truly intelligent diagnostic-therapeutic systems. Moreover, integration of ROS nanotechnology with complementary strategies to further enhance antibiotic uptake and reduce drug efflux warrants continued theoretical development and translational validation.

### ROS nanotechnology with immune activation for osteomyelitis treatment

The microenvironment of chronic osteomyelitis is often represented by a significant immunosuppressive state, manifested by immune cell dysfunction, imbalance between pro-inflammatory and anti-inflammatory signaling, and impaired immune surveillance 174. Immune dysregulation within bone marrow lesions not only weakens the host's ability to eradicate pathogens but also leads to therapeutic challenges such as restricted antibiotic penetration and aggravated biofilm formation, thereby representing a central mechanism underlying persistent infection and disease recurrence. As a key mediator of innate immune, ROS serve not only as potent antimicrobial effectors but also as regulators of immune homeostasis within the local microenvironment through regulation of immune cell activation and polarization states. Under conditions of high pathogen load, the dynamic immunomodulatory properties of ROS provide a promising strategy for reversing local immunosuppression and restoring effective host defense mechanisms 175.

Accordingly, application of ROS-based nanotechnology offers and restoring effective host defense mechanisms antibacterial efficacy and modulate the immunosuppressive microenvironment of osteomyelitis. Through effective pathogen eradication, ROS nanoplatforms reduce immune exhaustion, while concurrently promoting conversion of the local microenvironment from a state of immune tolerance to immune activation by activating innate immune responses and restoring immune cell functionality. This dual-mode regulatory mechanism transcends the limitations of conventional antimicrobial therapy and provides both theoretical foundation and technical support for reestablishing dynamic equilibrium between host immune defense and pathogen clearance. The following section first summarizes the immunological characteristics of the osteomyelitis microenvironment, followed by critical analysis of the advantages, limitations, and future development directions of existing ROS nanotechnology-based immune activation strategies, thereby offering theoretical guidance for integrated osteomyelitis treatment paradigms that combine ROS nanotechnology with immunotherapeutic approaches.

#### Characteristics of the immune microenvironment at the osteomyelitis site

##### Immune characteristics of acute osteomyelitis

The immune microenvironment of acute osteomyelitis is predominantly characterized by extensive neutrophil infiltration, elevated release of pro-inflammatory mediators, and activation of the complement system. During the early phase of infection, rapid recruitment of neutrophils to the affected site occurs, followed by phagocytic elimination of invading microorganisms. Concurrently, microbial invasion triggers both local and systemic inflammatory responses, resulting in increased production of pro-inflammatory cytokines, such as tumor necrosis factor-α (TNF-α) and interleukin-1β (IL-1β). These cytokines enhance vascular permeability and promote further recruitment of immune cells to the infected region. In parallel, activation of the complement cascade facilitates pathogen clearance and amplifies inflammatory signaling, collectively contributing to the acute immune defense response 176,177.

##### Immune characteristics of chronic osteomyelitis

During the chronic stage of osteomyelitis, the immune microenvironment is primarily characterized by sustained activation of macrophages and T lymphocytes. As infection persists, macrophages progressively become the dominant effector immune cells. Classically activated M1 macrophages play a central role in initiating and maintaining pro-inflammatory responses by secreting large amounts of pro-inflammatory cytokines, thereby facilitating pathogen clearance. Concurrently, M1 macrophages promote differentiation of T helper 1 (Th1) and T helper 17 (Th17) cells, further amplifying immune activation. M1 macrophage polarization is induced by pro-inflammatory stimuli such as interferon-γ (IFN-γ) and lipopolysaccharide (LPS) through the classical activation pathway 178-180. These signals typically arise from microbial invasion, tissue injury, or activation of immune cells including T lymphocytes and natural killer (NK) cells. Phenotypically, M1 macrophages exhibit elevated surface expression of CD80, CD86 and major histocompatibility complex (MHC) class II molecules, which enhance antigen presentation and reinforce pro-inflammatory immune responses. In addition, M1 macrophages secrete chemokines that recruit other immune cells, such as neutrophils and dendritic cells (DCs), to sites of infection 180,181. However, while M1-mediated responses contribute to effective pathogen clearance during early infection, prolonged M1 activation promotes persistent inflammation and progressive tissue damage, which is particularly evident in chronic inflammatory and autoimmune conditions.

Alternatively activated M2 macrophages play an important role in inhibiting inflammatory responses, promoting wound healing, and maintaining immune homeostasis 182. M2 polarization is induced primarily by anti-inflammatory cytokines, such as interleukin-4 (IL-4), interleukin-13 (IL-13), and interleukin-10 (IL-10). During later stages of the immune response, M2 activation contributes to resolution of inflammation and facilitation of tissue repair 182,183. Phenotypically, M2 macrophages express high levels of CD163 and CD206, markers associated with phagocytic clearance of damaged tissue and promotion of tissue remodeling. In addition, M2 macrophages secrete substantial amounts of anti-inflammatory mediators, such as IL-10 and transforming growth factor-β (TGF-β), which play essential roles in dampening inflammation and accelerating tissue regeneration 182,184.

During the chronic phase of osteomyelitis, activation of T lymphocytes also contributes to host antimicrobial defense. CD4⁺ T helper cells differentiate into multiple subsets, including Th1, Th2, and Th17, based on cytokine secretion profiles, thereby orchestrating distinct immune response pathways. IFN-γ produced by Th1 cells enhances antimicrobial activity, whereas Th17 cells are closely associated with inflammatory and autoimmune responses. Persistent inflammatory stimulation may further promote collagen deposition and fibrotic scar formation, ultimately disrupting normal bone remodeling and metabolic processes 185,186.

#### ROS nanotechnology for osteomyelitis therapy through immune regulation

##### Mechanisms of immune regulation

Participation of ROS in shaping the bone marrow immune microenvironment represents a critical determinant of the therapeutic efficacy of ROS-based nanotechnologies for osteomyelitis management. As both endogenous and exogenous signaling mediators, ROS regulate innate and adaptive immune responses by modulating the activity of multiple immune cell populations, including macrophages, DCs, T lymphocytes, and NK cells. These regulatory effects are mediated through activation of key signaling pathways, including TNF-β, mechanistic target of rapamycin (mTOR), ERK, and intracellular calcium signaling cascades. During antibacterial responses, ROS exert dual functions by directly inducing microbial cytotoxicity while simultaneously orchestrating immune cell activation and polarization. Accordingly, systematic examination of ROS-mediated regulatory pathways across different immune cell subsets is essential for elucidating the mechanisms by which ROS nanotechnology modulates the pathological microenvironment of osteomyelitis and enhances therapeutic outcomes.

***ROS and macrophages***: Polarization of classically activated M1 macrophages is regulated by multiple intracellular signaling pathways, predominantly including the mitogen-activated protein kinase (MAPK)s/NF-κB and Janus kinase-signal transducer and activator of transcription (JAK-STAT) cascades 187. Members of the nicotinamide adenine dinucleotide phosphate oxidase (NOX) family, especially NOX2 and NOX4, are closely related to ROS generation and M1 macrophage polarization. Activation of NOX2/NOX4 enhances ROS production along the electron transport chain, which subsequently stimulates MAPK and NF-κB signaling, amplifies inflammatory responses, and ultimately drives monocytes differentiation toward M1 phenotype 188,189. In addition, Cotzomi-Ortega *et al.* found that ROS-dependent macrophage migration inhibitor (MIF) induces M1 polarization via paracrine signaling mechanisms 190. Zhou *et al.* further demonstrated that iron overload-induced ROS upregulates p53 acetylation, thereby facilitating M1 macrophage polarization 191.

***ROS and T cells***: Accumulating evidence demonstrates that ROS critically regulate T-cell proliferation, differentiation, apoptosis, and effector function. Low physiological levels of ROS are essential for effective T-cell activation. Endogenous ROS generation in T lymphocytes arises primarily from mitochondrial metabolism and NOX 192,193. Mitochondria-derived ROS (mROS), produced by respiratory chain complexes I, II, and III, are indispensable for T-cell activation and promote signaling through nuclear factor of activated T cells (NFAT), NF-κB, and AP-1, thereby influencing secretion of IL-2 and IL-4, and regulating subsequent T-cell proliferation and differentiation 194. The contribution of NOX-derived ROS to T-cell activation remains an area of ongoing investigation. Exogenous ROS enhance T-cell activation by stimulating T-cell receptor (TCR) signaling and reinforcing transcriptional programs mediated by NFAT, AP-1, and NF-κB 192,195. TCR engagement induces rapid production of H_2_O_2_ and •O_2_^-^, which subsequently modulate ERK signaling and the human apoptosis-related factor ligand (Fas ligand, FasL/CD95L) apoptotic pathway, respectively 193.

T cell differentiation involves antigen-driven activation of CD4⁺ T cells or CD8⁺ T cells into helper T cells (Th) and cytotoxic T lymphocyte (CTLS) subsets. ROS have been shown to promote differentiation of Th1, Th2, and Th17 cells 193-195. NOX activation increases intracellular ROS levels and promotes polarization toward Th1 and Th17 phenotypes 199. Furthermore, ROS-mediated activation of ERK1/2 signaling enhances IL-4 production, thereby promoting Th2 differentiation 200. Abimannan* et al.* demonstrated that oxidative stress induced by pro-oxidants such as plumbagin and H_2_O_2_ upregulates Th1 and Th17 differentiation through enhanced ERK1/2-dependent oxidative phosphorylation 201. Kim *et al* further identified a pivotal role for ROS in regulating inflammatory functions of natural killer T (NKT) cells, a unique lymphocyte subset sharing features of both T cells and NK cells. ROS signaling promoted differentiation toward NKT1 and NKT17 subtypes while inhibiting NKT2 differentiation 202.

In addition to regulating activation and differentiation, ROS also contribute critically to T cell activation-induced cell death (AICD), a specialized apoptotic process that maintains immune homeostasis following T-cell activation. ROS-mediated AICD is primarily regulated through the Fas ligand (FasL) pathway and the ERK signaling pathway 203,204. TUpon TCR engagement, H_2_O_2_ production is initially catalyzed by dual oxidase-1 (Duox-1), which amplifies early TCR signaling. Sustained TCR activation subsequently promotes mitochondrial ·O_2_^-^ generation, leading to increased FasL expression on T cells and triggering Fas-dependent apoptotic signaling. Concurrently, TCR stimulation elevates intracellular H_2_O_2_ levels and activates ERK 1/2 phosphorylation, thus promoting apoptotic cascades and execution of AICD 205.

***ROS and neutrophils***: ROS play a central role in regulating neutrophil antimicrobial activity and programmed cell death 203,204. Upon pathogen invasion, neutrophils are rapidly recruited to infected tissues under the guidance of chemokines and eliminate pathogens primarily through phagocytosis. Internalization of microorganisms triggers a robust "respiratory burst”, characterized by a sharp increase in O_2_ consumption and massive ROS production 208. This process is driven by the high expression and activation of NADPH oxidase 2 (NOX2) in neutrophils, which constitutes one of the most powerful antimicrobial mechanisms of innate immunity 209. Within phagosomes, myeloperoxidase (MPO) and other antibacterial enzymes utilize H_2_O_2_ and halide or pseudo-halide ions to generate highly cytotoxic oxidants, including HOCl, HOBr, and hypothiocyanous acid (HOSCN). These oxidants induce extensive lipid peroxidation and oxidative DNA damage in engulfed pathogens, ensuring efficient microbial killing 210.

Apoptosis represents the predominant non-lytic death pathway of neutrophils and is tightly regulated by NOX-derived ROS 211. Scheel-Toellner *et al.* proved that NOX-generated ROS activate acidic sphingomyelase, promoting clustering of death receptors such as CD95 (Fas) and subsequent caspase-8 activation, ultimately driving neutrophil apoptosis 212. Conus *et al.* further confirmed that ROS facilitate the release of cathepsin D from lysosomes, which then activates caspase-8 and reinforces apoptotic signaling 213. Beyond apoptosis, NOX-regulated ROS production also critically influences neutrophil pyroptosis and necroptosis. Ryu *et al.* found that NOX2 deficiency reduces ROS levels, leading to aberrant activation of the P2X7 receptor-dependent noncanonical inflammasome pathway, culminating in enhanced neutrophil pyroptosis 214. Collectively, these findings establish ROS as pivotal regulators of both neutrophil antimicrobial function and fate determination within infected tissues.

***ROS and B cells*:** ROS play a critical regulatory role in B-cell maturation, activation, differentiation, and programmed cell death. B cells originate from pluripotent hematopoietic stem cells in the bone marrow and undergo a tightly regulated developmental program comprising pre-B cells, immature B cells, mature B cells, activated B cells, and plasma cells 215, 216. The early differentiation of pre-B and immature B cells is antigen-independent and occurs primarily within the bone marrow microenvironment 217. Accumulating evidence indicates that ROS are integral to B-cell receptor (BCR) signaling and B-cell activation. ROS modulate the phosphorylation status of BCR-associated kinases, thereby influencing downstream signaling cascades essential for B-cell activation. Upon antigen engagement, mature B cells undergo profound metabolic reprogramming characterized by increased mitochondrial biogenesis and PI3K-dependent glucose uptake, which in turn fuels the pentose phosphate pathway, providing reducing equivalents for oxidative stress control and substrates for nucleotide synthesis required during clonal expansion. Vené *et al* reported that ROS depletion significantly impairs BCR-mediated B-cell activation and proliferation, highlighting the indispensable role of ROS in sustaining B-cell immune responses 218. Similarly, Yang *et al.* demonstrated that sustained NOX-dependent ROS production following BCR stimulation enhances the activation of the NF-κB and AKT signaling pathways, thereby promoting B-cell proliferation and survival 219.

B cells can be further classified into B1 and B2 subpopulations, which originate from distinct hematopoietic precursors: B1 cells primarily derive from fetal liver-derived hematopoietic stem cells (HSCs), whereas B2 cells originate from bone marrow-derived HSCs 220. During B-cell differentiation, endoplasmic reticulum-derived ROS are greatly amplified, a process that supports efficient immunoglobulin (Ig) synthesis and secretion. Moreover, ROS generated through mitochondrial depolarization play a crucial role in promoting the differentiation of activated B cells into long-lived memory B cells, thereby sustaining humoral immune memory 221.

Apoptosis, the predominant mode of B-cell death, is tightly regulated by intracellular ROS levels. Physiological (low) ROS concentrations are indispensable for maintaining B cell activation and function, whereas excessive ROS accumulation triggers oxidative stress, leading to cellular injury and programmed cell death. Mechanistically, elevated ROS levels activate caspase-9, leading to upregulation of XAF1, which subsequently induces apoptosis in EBV-transformed B cells. In parallel, ROS activate the JNK/p38-MAPK signaling pathway, promoting mitochondrial translocation of Bax, disruption of mitochondrial membrane potential, and subsequent activation of caspase-9 and caspase-3 222. In addition to apoptosis, ROS also contribute to pyroptotic B-cell death. ROS-mediated activation of inflammasomes, including NLRP3, NLRP6, NLRC4, and AIM2, enhances caspase-1-dependent pyroptosis, further influencing B-cell survival and immune homeostasis 223.

***ROS and DCs***: As specialized antigen-presenting cells, DCs play a central role in initiating immune responses, regulating immune homeostasis, and maintaining immune tolerance. Immature DCs undergo activation and maturation to acquire potent antigen-processing and T-cell-priming capacity. Consequently, precise regulation of DCs immune function is crucial for the treatment of immune-related disorders, including autoimmune diseases, infections, and cancer. Accumulating evidence indicates that ROS significantly affects the differentiation, activation, and functional programming of DC subsets, including monocyte-derived DCs (MoDCs) and plasmacytoid DCs (pDCs) 224. For MoDCs, maintenance of physiological ROS levels is essential for their activation, maturation, migration, cytokine secretion, and T-cell stimulatory capacity. Peng *et al.* revealed that ROS depletion inhibits the double-stranded RNA-dependent protein kinase (PKR), protein kinase C (PKC), and p38/MAPK signaling pathways, resulting in downregulation of NF-κB, co-stimulatory molecules (CD40, CD80, CD86), and pro-inflammatory cytokines ( IL-1β, TNF-α and IL-12) 225. Complementary studies have shown that ROS elevation—induced by the xanthine-xanthine oxidase system or cationic liposomes—promotes MoDC activation and maturation. Cheong *et al.* further found that increased ROS enhances MoDC maturation via phosphorylation of p38, JNK, and ERK, accompanied by nuclear translocation of NF-κB, thereby augmenting the capacity of MoDCs to activate allogeneic CD8^+^ T cells and CD4^+^ Th1 cells, while concurrently inhibiting Th2 differentiation 226. For pDCs, ROS homeostasis is likewise crucial for their activation and immune function. Oberkampf* et al.* reported that ROS downregulation significantly inhibits the expression of CD69, CD40, CD80, and CD86, and the secretion of IFN-α, underscoring the necessity of ROS for normal pDC physiology 227. Moreover, while elevated ROS does not markedly alter Th1 and Th17 differentiation induced by resting pDCs, it significantly enhances Th2 polarization, indicating that ROS exerts context-dependent regulatory effects on T-cell differentiation mediated by pDCs 228.

***ROS and NK cells***: NK cells are cytotoxic lymphocytes lymphoid progenitors and constitute a critical component of the innate immune system, providing rapid first-line defense against invading pathogens and malignant cells. Increasing evidence indicates that ROS levels exert a tightly regulated, bidirectional influence on NK cell survival and effector function. While physiological, low-level ROS are necessary to sustain NK cell viability and functional competence, excessive oxidative stress severely compromises NK cell activity. Harmon *et al.* verified that extracellular acidification induces mitochondrial dysfunction in NK cells, resulting in elevated intracellular ROS accumulation, which in turn triggers NK cell apoptosis and impairs antitumor immune responses. Conversely, activated NK cells actively suppress intracellular ROS to preserve their cytotoxic function. For example, IL-15-induced NK cells up regulate the thioredoxin antioxidant system, effectively reducing intracellular ROS levels and protecting NK cells from oxidative damage. These findings highlight a dynamic, bidirectional regulatory relationship between ROS and NK cells, in which balanced redox homeostasis is essential for maintaining NK cell-mediated immune surveillance and host defense 229.

##### Application of ROS nanotechnology for immune regulation

ROS play an important role in both the physiological function and immune regulation of bone marrow cells, making ROS nanotechnology a powerful tool for reshaping the immune microenvironment in osteomyelitis therapy. First, ROS directly exert potent antimicrobial effects through oxidative damage to microbial membranes, proteins, and nucleic acids. Simultaneously, ROS participate in the dynamic regulation of inflammatory responses by modulating macrophage polarization. By activating signaling pathways such as AMPK, ROS can promote the transition of monocytes toward the pro-inflammatory M1 phenotype during early infection and subsequently facilitate the shift from M1 to M2 macrophages during later stages of tissue repair, thereby orchestrating both pathogen clearance and wound resolution. Second, ROS act as key signaling mediators that bridge innate and adaptive immunity. Elevated ROS levels at infection site induce Immunogenic cell death (ICD) of pathogens and damaged host cells, generating abundant antigenic signals that stimulate immune activation. During ICD, multiple danger-associated molecular patterns and cytokines are released, including membrane-associated signaling molecules and pro-inflammatory mediators, which collectively amplify immune surveillance. Moreover, ROS directly promote the activation and maturation of DCs by triggering intracellular signaling pathways, including TNF-β, mTOR, ERK, and calcium signaling cascades. Importantly, ROS facilitate efficient antigen processing and presentation by enhancing lysosomal escape of internalized antigens and protecting them from degradation, thereby improving cytoplasmic antigen delivery. This process significantly strengthens antigen cross-presentation and induces robust CD8⁺ T-cell responses, further reinforcing antimicrobial immunity. Through the coordinated regulation of antimicrobial activity, immune cell activation, inflammatory resolution, and tissue regeneration, ROS-based therapeutic strategies generate profound immune feedback that critically determines overall outcome of osteomyelitis treatment.

**Figure 9:**
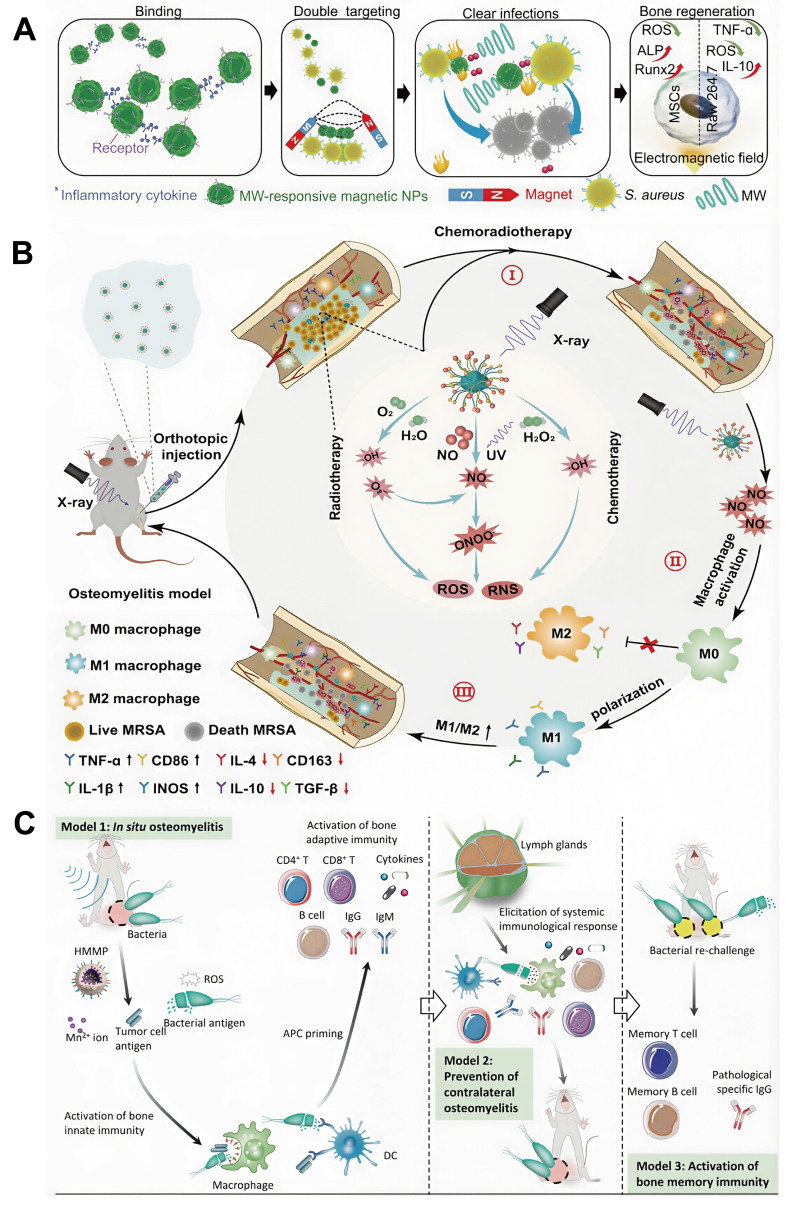
(A) Schematic illustration of the fabrication process of engineered pseudo-macrophage nanoparticles and the biological property and characterization of the microwave-responsive nanoplatform. Adapted with permission from Ref. 230. Copyright 2021, Wiley-VCH. (B) Schematic design of the SNP nanoradiosensitizer and its anti-biofilm and immunomodulatory functions for osteomyelitis therapy. Adapted with permission from Ref. 232. Copyright 2024, Wiley-VCH. (C) Schematic representation of HMMP-mediated *in situ* nanovaccination for immune activation in osteomyelitis treatment. Adapted with permission from Ref. 99. Copyright 2022, American Chemical Society.

In view of the pivotal roles of M1 and M2 macrophages in antimicrobial defense and wound healing, respectively, macrophage-targeted ROS nanomaterials have been actively developed for osteomyelitis therapy. Fu *et al.* constructed a microwave-responsive engineered pseudo-macrophage-coated Fe_3_O_4_/Au nanoparticle (M-Fe_3_O_4_/Au), which weakened inflammatory responses and induced M2 macrophage polarization within osteomyelitis lesions by neutralizing pro-inflammatory cytokines and reducing intracellular ROS production 230 (**Figure 9A**).

Similarly, Cai *et al.* developed a novel US-responsive heterojunction array p-ZnO/TiO_2_-x, of which nanorod morphology activated the Fn-Integrin-α5β1-PI3K-AKT1 signaling pathway in macrophages, thereby inducing M1-to-M2 polarization and alleviating local inflammation, as evidenced by increased IL-10 levels and decreased IL-1β expression in surrounding tissues 231. In parallel, ROS nanomaterials capable of promoting M1 macrophage polarization have also been engineered to enhance antibacterial efficacy. Zhang *et al.* integrated a PEG-based photosensitive nitric oxide donor (PEG-b-pCouNO) with an antimicrobial peptide (Nisin) onto Cs_3_Cu_2_I_5_ nanoscintillators, forming Cs_3_Cu_2_I_5_@(Nisin+PEG-b-pCouNO) nanoparticles (SNP NPs). Upon activation, PEG-b-pCouNO released NO and depleted intracellular GSH, thereby relieving tissue hypoxia and enhancing ROS production. Concurrently, NO signaling drove the polarization of immunosuppressive M0 macrophages toward the pro-inflammatory M1 phenotype. This synergistic strategy of combining immunomodulation with chemo-radiotherapeutic augmentation, showed highly effective therapeutic outcomes in MRSA-infected osteomyelitis 232 (**Figure 9B**).

In addition, macrophage polarization, the exploration of other immune activation pathways provides important support for the development of next-generation immunotherapeutic strategies for osteomyelitis. For example, Lin *et al.* developed a biomimetic nanotherapeutic platform termed HMMP, in which PpIX was encapsulated within hollow MnOx nanoparticles and subsequently cloaked with a hybrid membrane derived from macrophages and tumor cell lines. This multifunctional system not only activated innate immunity responses to enhance direct pathogenic bacterial clearance, but also stimulated adaptive immunity through antigen burst release and subsequent APC activation. Importantly, the resulting activation of immune memory effectively prevented infection recurrence, demonstrating durable therapeutic protection in osteomyelitis models 99 (**Figure 9C**).

Therefore, the optimization of chronic osteomyelitis treatment fundamentally depends on overcoming the dual barriers of pathogen persistence and local immunosuppression. Conventional antimicrobial strategies predominantly focus on microbial eradication while largely neglecting immune microenvironment remodeling. In contrast, ROS-responsive nanotechnology enables the spatiotemporally controlled integration of antibacterial effect and immune activation, thereby establishing a synergistic therapeutic paradigm for osteomyelitis management. This approach not only introduces an innovative "antibacterial and immunomodulatory" strategy for refractory bone infections, but also underscores the importance of restoring host-pathogen homeostasis as a central objective in future anti-infective therapies. By integrating materials engineering with immune regulation, these intelligent delivery systems define a new paradigm for targeted immunometabolic intervention, with broad translational potential for the treatment of chronic infections and immune dysregulation-associated diseases.

### ROS nanotechnology with bone repair for osteomyelitis treatment

Chronic osteomyelitis is usually accompanied by varying degrees of bone defects. During the self-repair process, adequate new bone formation is necessary for the reconstruction of skeletal structure and function. The difficulty of bone defect repair is closely related to defect size. When the defect area exceeds the intrinsic osteogenic healing capacity, rapidly migrating fibrous connective tissue occupies the defect site before osteoblast infiltration, while persistent bacterial infection further compromises bone regenerative potential, ultimately leading to adverse clinical sequelae 233. Currently, conventional management of extensive osteomyelitis-associated defects relies on infection control using systemic or local antibiotics combined with defect reconstruction via autografts or allografts. However, such strategies are often limited by prolonged treatment duration, incomplete eradication of infection, and suboptimal regenerative outcomes. In response to these limitations, numerous novel nanobiomaterials possessing both ROS-mediated antibacterial activity and osteoinductive capacity have recently been developed for osteomyelitis treatment. The following section systematically delineates the molecular mechanisms underlying bone metabolic imbalance during osteomyelitis, examines latest advances in ROS-based nanotechnologies for reconstruction of the bone regeneration microenvironment, and critically discusses the advantages, limitations, and future directions of the emerging “antibacterial-anti-inflammatory-osteogenic" trinity therapeutic paradigm, thereby providing theoretical guidance for integrated osteomyelitis treatment strategies combining ROS nanotechnology with bone repair approaches.

#### Mechanism of bone loss in osteomyelitis progression

**Figure 10:**
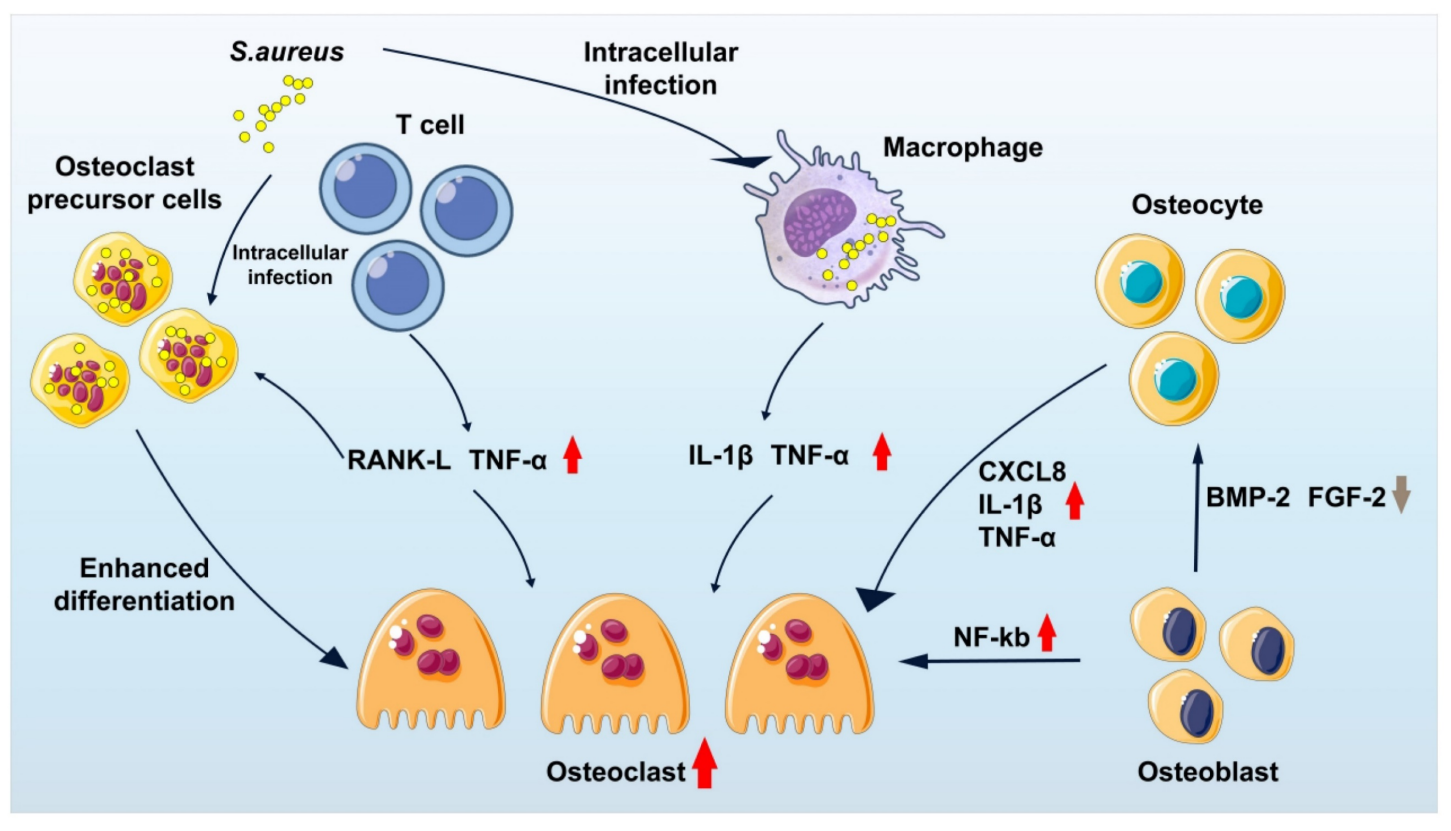
Schematic illustration of inflammation-induced imbalance in the bone immune microenvironment during osteomyelitis.

When bone tissue is invaded by bacteria, the host immune system is rapidly activated and releases a broad spectrum of inflammatory mediators and cytokines to eliminate invading pathogens. However, these inflammatory factors exert dual pathological effects on bone metabolism. On the one hand, they significantly enhance osteoclast differentiation and activity, thereby accelerating bone resorption; on the other hand, they simultaneously suppress osteoblast function, impairing new bone formation and regeneration 174 (**Figure 10**). The combined imbalance between excessive bone resorption and insufficient bone formation ultimately leads to progressive bone loss and structural destruction at osteomyelitis lesions. In this section, the specific molecular and cellular mechanisms through which increased osteoclast activity and decreased osteoblast function contribute to bone loss during osteomyelitis progression are discussed in detail, providing a theoretical basis for the development of targeted therapeutic strategies.

##### Enhanced osteoclast activity

Osteoclasts are multinucleated cells derived from the monocyte-macrophage lineage that mediate bone resorption through the secretion of proteolytic enzymes and H^+^ ions. Osteoclasts activity is tightly regulated by immune cells, including macrophages, neutrophils, and T lymphocytes.

**Macrophage and osteoclast activity**: Upon pathogenic stimulation, macrophages markedly up regulate pro-inflammatory factors to fight infection and initiate immune responses. In particular, macrophage-derived TNF-α and IL-1β aggravate osteocyte apoptosis. Apoptotic osteocytes subsequently release receptor activator of nuclear factor-κB ligand (RANKL), a key signaling molecule that drives osteoclast proliferation and differentiation 234. This cascade promotes excessive osteoclast accumulation at the site of infection and accelerates pathological bone destruction. Furthermore, during *S. aureus* invasion of bone marrow, macrophages activate he NOD-like receptor pyrin domain-containing protein 3 (NLRP3) inflammasome, leading to caspase-1 activation and enhanced IL-1β maturation, which further stimulates osteoclastogenesis via the c-Jun JNK and NF-κB signaling pathways 235.

**Neutrophil and osteoclast activity**: Neutrophils are highly motile phagocytes that constitute the earliest cellular responder during microbial invasion. Following bacterial infiltration of bone marrow, chemokines produced by host cells and pathogens rapidly recruit neutrophils from the bloodstream, facilitating transendothelial migration toward the infected site. Experimental studies have demonstrated that, upon *S. aureus* infection of bone tissue, infiltrating neutrophils secrete a large number of inflammatory cytokines, such as IL-6, which activate the NF-κB signaling pathway and promote differentiation of osteoclast precursors into mature bone-resorbing osteoclasts 26. Moreover, sustained neutrophil accumulation within inflamed bone tissues produces excessive levels of ROS, nitric oxide synthase products, and neutrophil extracellular traps (NETs). This oxidative overload disrupts redox homeostasis, trigger aberrant inflammatory responses, and ultimately induces cytotoxicity, tissue injury, and progressive bone loss 195.

**T cell and osteoclast activity**: Within infected bone marrow, activated macrophages exhibiting elevated expression of IFN-α and IL-12 activate NK cells and induce the differentiation of CD4⁺ T cells toward the Th1 lineage. Although Th1 cells contribute to pathogen clearance through secretion of IFN-γ and TNF-α, the concomitant upregulation of RANKL and TNF-α constitutes a major stimulus for osteoclast differentiation. In parallel, IL-6 and transforming growth factor-β (TGF-β) promote maturation of Th17 cells and upregulate IL-17 production, which further increases RANKL expression in both osteoclast precursors and osteoblasts, thereby accelerating osteoclastogenesis and bone resorption 174.

**Other elements and osteoclast activity**: Beyond immune cell regulation, multiple additional factors participate in controlling osteoclast activity during osteomyelitis. Toxic shock syndrome toxin-1 (TSST-1), a superantigen produced by *S. aureus* to suppress host immunity, has been shown to directly enhance the bone-resorptive capacity of osteoclasts 37. Trouillet-Assant *et al.* confirmed that, compared with osteoclast precursors, mature osteoclasts show a significantly greater capacity to internalize *S. aureus*, which in turn promotes osteoclast fusion and further augments bone resorption 236. Moreover, Somayaji *et al.* found that osteoblasts infected with *S. chrysosa* significantly upregulate the prostaglandin E2 (PGE2) production, a potent activator of osteoclastic bone resorption mediated via the RANKL-dependent signaling pathway 237.

Collectively, osteoclast activity is governed by a complex network of immune mediators, microbial virulence factors, and bone-derived signals. Within the inflammatory microenvironment of osteomyelitis, this regulatory balance becomes severely disrupted, resulting in excessive osteoclast activation and accelerated bone loss. Targeting key regulatory pathways involved in osteoclast differentiation and function therefore represents a promising therapeutic strategy to mitigate bone loss and preserve skeletal integrity during osteomyelitis progression.

##### Decreased osteoblast activity

*S. aureus* infection of bone tissue leads to robust production of chemokines, such as CXCL8, CXCL9, and CXCL10. Among these mediators, CXCL8 significantly upregulates the release of IL-1β and TNF-α. Elevated TNF-α subsequently activates t NF-κB signaling in osteoblasts while simultaneously inhibiting the expression of key osteogenic regulators, such as BMP-2 and fibroblast growth factor 2 (FGF-2). In addition, CXCL8 promotes neutrophil chemotaxis and upregulates the expression of matrix metalloproteinases (MMP), which actively participate in extracellular matrix degradation and bone resorption 235. Consequently, osteoblast dysfunction constitutes a critical contributor to impaired bone regeneration and progressive skeletal destruction during osteomyelitis. Preservation or restoration of osteoblast activity therefore represents an essential therapeutic objective for effective bone repair and structural reconstruction. Targeted regulation of inflammatory signaling and osteogenic factor expression offers a promising intervention for preventing pathological bone loss and improving long-term skeletal outcomes in osteomyelitis.

#### ROS nanotechnology for osteomyelitis therapy by promoting bone repair

ROS contribute to bone regeneration through multiple interconnected mechanisms, including modulation of the immune microenvironment, stimulation of angiogenesis, initiation of cellular proliferation and differentiation, and regulation of intracellular signaling pathways. First, controlled levels of ROS provide essential exogenous stimuli that enhance macrophage activity within the hyperglycemic and immunosuppressive microenvironment characteristic of chronic osteomyelitis. ROS promote macrophage metabolic reprogramming by activating glycolytic and glucose metabolism pathways, thereby strengthening the reparative function of M2 macrophages during bone healing 238. Activated M2 macrophages subsequently establish a pro-regenerative milieu by secreting anti-inflammatory cytokines and angiogenic factors that support tissue reconstruction 239. Second, ROS regulate vascular endothelial cell function at bone defect sites by activating the PI3K/Akt signaling pathway, thereby stimulating neovascularization, which is indispensable for nutrient delivery and metabolic waste clearance during bone regeneration 240. Moreover, ROS influence local oxidative phosphorylation and Wnt signaling pathways, indirectly contributing to vascular remodeling and tissue integration 241. Additionally, transient elevation of ROS during the early phase of tissue damage activates apoptotic signaling and initiates osteoprogenitor regeneration via compensatory proliferation mechanisms 242. Physiological concentrations of ROS further participate in directing osteogenic differentiation of mesenchymal stem cells and directly promote extracellular matrix deposition and bone formation 243. Finally, as key second messengers, ROS orchestrate redox-sensitive signaling networks that govern bone development and remodeling by coordinating the functional activities of mesenchymal cells, osteoblasts, osteoclasts, and endothelial cells, thereby maintaining dynamic skeletal homeostasis 244.

These findings collectively highlight the significant therapeutic potential of ROS in bone repair. Future development of ROS-based nanoplatforms is expected to enable precise loading and controlled release of osteogenic agents, thereby improving local drug bioavailability and therapeutic efficacy. Such nanoplatforms not only stimulate the secretion of osteogenic factors but also accelerate bone formation by activating key osteogenic signaling pathways. Moreover, ROS nanotechnology can suppress osteoclast activity and effectively attenuate excessive bone resorption, contributing to the preservation of bone mass and structural stability. Consequently, ROS-based nanotherapeutic systems establish a synergistic “antibacterial-bone regenerative" treatment paradigm that simultaneously eradicates infection while promoting functional bone regeneration and tissue.

##### ROS nanotechnology for osteomyelitis therapy by loading osteogenic drugs

Integration of ROS nanotechnology with osteogenic drug delivery enables simultaneous antimicrobial therapy and bone regeneration at osteomyelitis lesions. Dexamethasone (Dex), a well-established osteogenic inducer capable of promoting osteogenic differentiation and bone formation in mesenchymal stem cells, has been widely explored in this context. Wu *et al.* developed a multifunctional nano system by co-loading antibacterial silver (Ag) nanoparticles and Dex into polydopamine-functionalized mesoporous silica nanoparticles (MSNs), which were subsequently incorporated into a poly-L-lactic acid (PLLA) scaffold for osteomyelitis treatment. The resulting Ag-pMSNs@Dex/PLLA nanosystem exhibited efficient ROS generation and significantly enhanced activation of bone marrow mesenchymal stem cells (BMSCs), thereby promoting robust bone regeneration while maintaining strong antibacterial efficacy 245.

##### ROS nanotechnology for osteomyelitis therapy by inducing osteogenic factors

**Figure 11:**
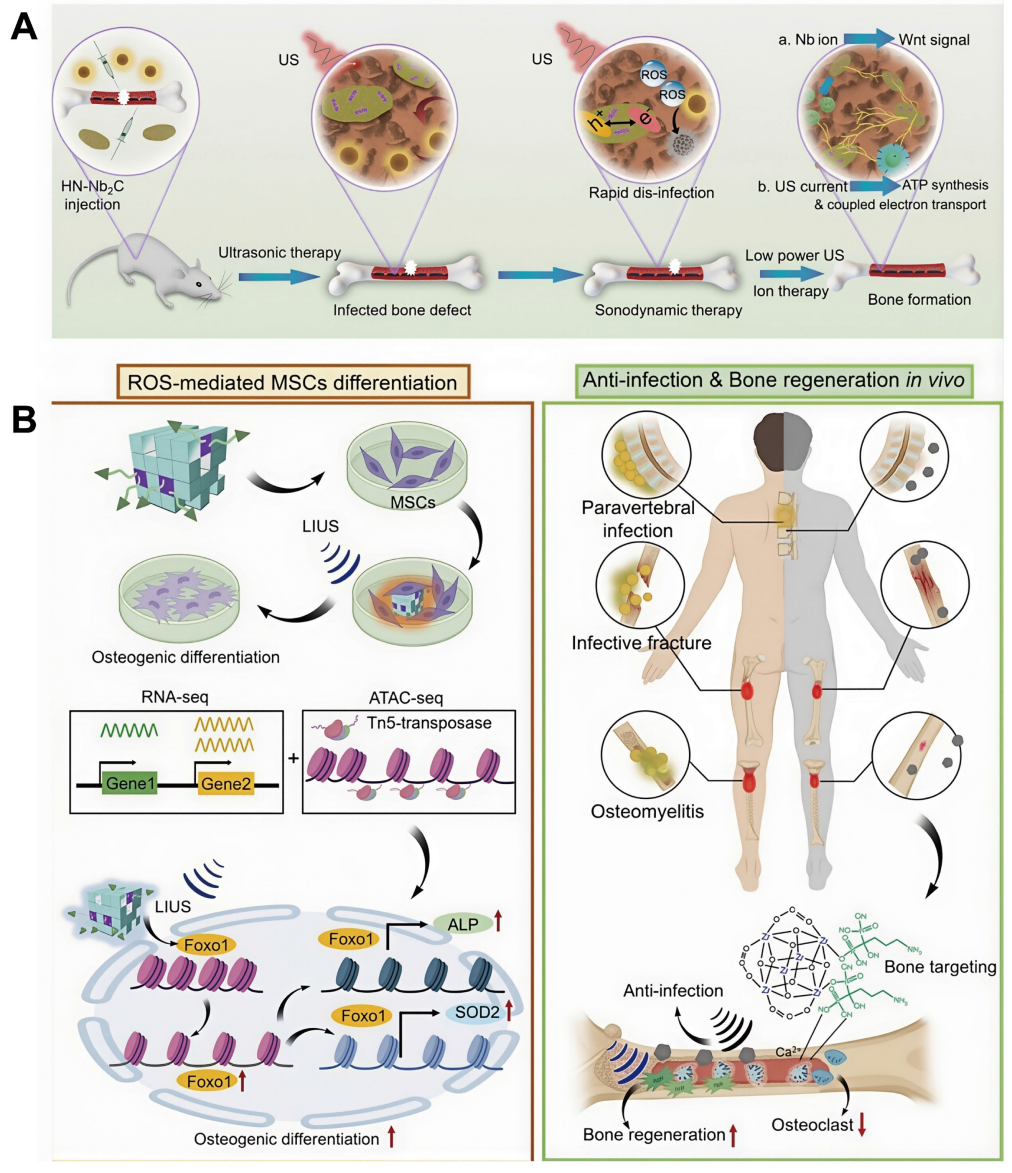
(A) Proposed mechanism underlying sonodynamic antibacterial treatment and ROS-mediated bone regeneration. Adapted with permission from Ref. 246. Copyright 2023, Springer Nature. (B) Schematic illustration of ALN-mediated defect-engineered MOF for targeted repair of infected bone tissue through sono-epigenetic modulation of chromatin accessibility. Adapted with permission from Ref. 247. Copyright 2024, Wiley-VCH.

ROS-based nanotechnology can further enhance bone regeneration by upregulating osteogenic factor expression and activating osteogenic signaling pathways. Ma *et al.* designed a Nb_2_C nanosheet modified porphyrin metal-organic frame hollow nanotubes (HNTM/Nb_2_C), which not only stimulated BMSCs through controlled ROS production and Nb^2+^ ion release but also significantly increased the expression of osteogenic genes, such as BMP2 and RUNX2, thereby promoting osteogenic differentiation and bone formation 246 (**Figure 11A**).

##### ROS nanotechnology for osteomyelitis therapy by inhibiting osteoclast activity

Bone tissue is mainly composed of nano-hydroxyapatite and organic matrix, and the structural characteristics of hydroxyapatite critically influence the osteogenic microenvironment and subsequent bone regeneration. Alendronate sodium (ALN), a clinically approved bisphosphonate, selectively binds bone mineral hydroxyapatite and inhibits osteoclast-mediated bone repair. Leveraging this mechanism, Ma *et al.* constructed a defect-engineered porphyrin-based MOF acoustic sensitizer (HN25) capable of simultaneously delivering ALN. The HN25 system regulated the bone marrow microenvironment via acoustodynamically mediated ROS generation and promoted bone repair by increasing chromatin accessibility of osteogenesis-related genes and forkhead box protein O1 (FOXO1_ 247 (**Figure 11B**).

Beyond direct antibacterial activity, the combined effects of reversing immunosuppression during remodeling of the bone marrow abscess microenvironment and promoting bone repair constitute a major therapeutic advantage of ROS-based technologies for osteomyelitis treatment. Recent comprehensive reviews have extensively addressed novel delivery systems for antibiotics or osteogenic factors in bone defects, as well as the general immune responses elicited by infection. However, systematic investigations focusing on the ROS-mediated reconstruction of the specific immune landscape in osteomyelitis—such as macrophage polarization, DC activation, and T-cell regulation within infected bone marrow—and its synergistic integration with targeted bone regeneration strategies remain limited.

### ROS strategies in different types of osteomyelitis

Osteomyelitis represents a heterogeneous group of disorders of which clinical manifestations and therapeutic responses vary substantially according to underlying systemic conditions, including diabetes mellitus, peripheral vascular disease, and immunocompromised states. Among these, diabetic foot osteomyelitis (DFO) constitutes one of the most refractory forms, in which conventional treatment strategies frequently fail because of compromised blood perfusion, peripheral neuropathy, and impaired immune function. These pathological features not only accelerate disease progression but also severely limit drug delivery and host-mediated pathogen clearance, thereby necessitating disease-specific and mechanism-guided therapeutic approaches.

#### ROS strategies in diabetic foot osteomyelitis

ROS-based therapeutic strategies, including PDT, SDT, CDT, and MWDT, exhibit promising potential in overcoming the treatment challenges of DFO, particularly those associated with persistent infection and impaired tissue regeneration. PDT has demonstrated efficacy in diabetic foot infections by selectively targeting bacterial cells and biofilms within infected tissues. In addition, SDT has emerged as a potential modality for the management of biofilm-associated infections, which represent a hallmark of chronic DFO. US-activated nanomaterials generate ROS upon acoustic stimulation, leading to the disruption of antibiotic-resistant biofilms and enhancing bacterial eradication. Furthermore, the superior tissue penetration capacity of US compared with light enables SDT to effectively reach deep-seated osseous lesions, rendering this approach particularly suitable for the treatment of advanced DFO involving deeper bone structures.

#### ROS strategies in other types of osteomyelitis

The application of ROS-based therapeutic modalities extends beyond DFO to encompass osteomyelitis resulting from trauma, open fractures, and post-surgical infections, conditions that are often characterized by complex polymicrobial colonization and dense biofilm formation. In such refractory cases, CDT offers distinct advantages by generating highly cytotoxic ·OH that directly disrupt bacterial cell walls and biofilms matrices. Moreover, the oxygen-independent nature of CDT renders it particularly well suited for osteomyelitis lesions, which commonly exhibit hypoxic environment. MWDT provides additional advantages in anatomically complex regions, where the synergistic combination of localized hyperthermia and ROS-mediated antimicrobial activity is especially beneficial for the eradication of infections. The application of microwave-responsive nanomaterials enables deep tissue penetration and precise thermal control, facilitating effective treatment of chronic infections, including those associated with orthopedic implants and prosthetic devices.

## Challenges and prospects

The rapid development and expanding application of ROS-based nanomedicine in the biomedical field have created significant opportunities to improve therapeutic outcomes in osteomyelitis treatment. Nevertheless, translation of these promising technologies into routine clinical practice remains constrained by multiple scientific, technical, and regulatory challenges. The major limitations and future directions for ROS-based nanomedicine in osteomyelitis therapy are summarized as follows.

### Biosafety risks

The clinical translation of ROS-based nanotechnology remains limited by significant biosafety concerns. Many platforms employed in PDT, SDT, CDT, and MWDT incorporated metal components, raising the risk of long-term metal retention and accumulation within the body. Moreover, although ROS exhibit potent, non-drug-resistant antimicrobial activity, excessive ROS generation may induce oxidative stress and collateral damage in healthy bone tissue. Consequently, rigorous evaluation of the biosafety profile and metabolic behavior of ROS nanomaterials is particularly important for their clinical application in osteomyelitis treatment. While short-term toxicity and biodistribution of various ROS nanomaterials have been studied, long-term biological effects and latent safety risks remain insufficiently characterized. Future development should prioritize nanomaterials with highly biodegradability or efficient renal clearance to meet clinical safety requirements. Several key parameters require careful optimization. Particle size is widely recognized as a critical determinant of nonmaterial toxicity 248; therefore, precise control of nanoparticle size and morphology is necessary to minimize systemic toxicity, undesired organ accumulation, and excessive cellular uptake. In addition, positively charged ROS nanoparticles typically exhibit higher cytotoxicity than neutral counterparts due to enhanced membrane disruption and mitochondrial stress induction 249, suggesting that the modulation of surface charge represents an effective approach for reducing biological toxicity. Furthermore, rational surface modification and functionalization can substantially improve biocompatibility. For instance, Yu *et al.* reported that GSH encapsulation greatly enhanced renal clearance efficiency of fluorescent gold nanoparticles, thereby reducing systemic retention and toxicity 249.

### Elucidation of the specific mechanism underlying ROS therapy

Current studies of ROS-based therapies for osteomyelitis have predominantly emphasized therapeutic efficiency, whereas the underlying molecular mechanisms governing ROS-mediated antimicrobial and regenerative effects remain incompletely understood. Integration of computational chemistry with materials science offers a powerful strategy for elucidating these mechanisms at the molecular level. Emerging reports have demonstrated the potential of such interdisciplinary approaches to predict ROS-related reaction pathways, thereby guiding rational design and synthesis of novel redox-active nanomaterials while providing a theoretical framework for experimental verification 250. With continued advances in cheminformatics and molecular modeling, computational methodologies are expected to delivery increasingly detailed insights into ROS-driven therapeutic processes, facilitating mechanistic understanding the accelerating translation of ROS-based technologies into clinically effective osteomyelitis treatments.

### Design and synthesis of new ROS nanomaterials

#### Stability of nanomaterials

The global healthcare sector is rapidly advancing toward an era of precision medicine, imposing increasingly stringent requirements on the rational design and synthesis of ROS-based nanomaterials. Future research and development of ROS nanotechnology for osteomyelitis therapy should focus on the optimization of nanomaterials with well-defined structures, compositions, and surface properties. Such platforms must accommodate individualized therapeutic demands while exhibiting excellent biocompatibility, high targeting efficiency, and clearly characterized *in vivo* metabolic behavior. The therapeutic efficacy of ROS-based treatment relies heavily on precise targeting and efficient delivery of nanomaterials to infected bone tissues. However, *in vivo* transport of ROS nanoparticles remains constrained by multiple physiological barriers, including systemic circulation dynamics and immune-mediated clearance, which collectively limit delivery efficiency and targeting precision 251. Moreover, uncertainties surrounding *in vivo* biodistribution, mechanisms of action, and long-term biological effects necessitate continued investigation. Equally critical is the physicochemical stability of ROS nanomaterials throughout synthesis, storage, and clinical application. Development of effective stabilizing strategies is therefore indispensable for preserving nanoparticle integrity. A variety of stabilizers, including surfactants, silica coatings, biomolecules, polymers, and metal shells, have been employed to maintain structural stability and prevent aggregation or premature dissolution of nanoparticles, thereby enchanting their functional reliability in biomedical applications 252,253.

#### Design of intelligent nanocarriers

Future development of ROS-based nanotechnology should incorporate the concept of "intelligent nanocarriers" stop enable more precise and personalized therapeutic interventions. Integration of artificial intelligence (AI) and machine learning algorithms into nanomaterial design and delivery planning offers a powerful framework for optimizing therapeutic performance. Specifically, AI-driven modeling can rapidly identify optimal nanoparticle parameters—including particle size, surface functionalization, and delivery strategies—thereby simultaneously enhancing ROS production efficiency and minimizing safety risks. Moreover, AI-assisted analysis of patient-specific clinical data, such as infecting microbial strains, genetic profiles, and local blood perfusion characteristics, can inform individualized carrier design and delivery protocols, enabling truly personalized osteomyelitis treatment. When combined with high-throughput experimental screening and computational simulation, these approaches allow prediction of nanomedicine biodistribution and therapeutic efficacy across diverse patient populations, thereby accelerating translational development and improving clinical outcomes 254.

#### Design of multimodal treatment strategies

Strategic integration of disease-stage-specific requirements with spatiotemporally controlled multimodal ROS nanotechnology represents a promising therapeutic paradigm for osteomyelitis. Multimodal treatment strategies are anticipated to become a central direction in the future development of ROS-based therapies. In particular, combining ROS nanotechnology with complementary modalities such as immune modulation and genome-editing approaches, offers substantial potential for improving therapeutic outcomes in complex and refractory osteomyelitis.

ROS-based nano platforms have demonstrated the capacity to significantly enhance the efficacy of immunotherapy, thereby reinforcing antibacterial responses. This confirms the feasibility of dual-modality treatment strategies that combine ROS nanotechnology with immunotherapeutic interventions, especially in the treatment of chronic and drug-resistant osteomyelitis. However, the therapeutic synergy between ROS nanotechnology and immunotherapy remains constrained by local immunosuppression, immune tolerance, and bacterial immune evasion within the infection microenvironment. Accordingly, continued optimization of ROS -immunotherapy combination strategies represents a critical frontier in osteomyelitis treatment development. Emerging approaches, such as co-administration of ROS nanotechnology with immune checkpoint inhibitors, may further strengthen host immune recognition and clearance of pathogenic bacteria while mitigating immune escape mechanisms. Moreover, future integration of ROS-based systems with advanced immunotherapeutic technologies—including chimeric antigen receptor (CAR) T-cell therapy, gene-editing platforms, or cytokine-based therapies—may provide transformative solutions for refractory osteomyelitis management 255.

Gene-editing technologies, particularly CRISPR-Cas9, have become powerful tools for correcting gene mutations, regulating gene expression, and intervening in disease-related molecular pathways 256. Integration of ROS-based nanotechnology with gene-editing approaches is expected to enhance localized ROS generation and amplify immune activation within infected tissues. Genetically engineered immune cells exhibit increased sensitivity to pathogenic microorganisms, thereby facilitating bacterial clearance and inhibiting immune evasion. However, current applications of gene-editing therapies remain constrained by limited delivery efficiency, potential off-target effects, and suboptimal cellular stability. Next-generation CRISPR systems with improved precision and specificity, such as CRISPR-Cas12 and CRISPR-Cas13 platforms, are expected to provide more clinically viable alternatives for therapeutic gene modulation in the future 257.

#### Clinical translation challenges and potential of ROS technology

Although ROS-based nanotechnology has shown remarkable efficacy in immune activation and eradication of drug-resistant bacteria, its clinical translation remains constrained by potential toxicity to host tissues. Excessive ROS production may induce oxidative stress, tissue injury, and undesirable inflammatory responses. Therefore, precise spatiotemporal control over generation, both in damage and duration, in essential for ensuring therapeutic safety and efficacy. Equally important is the targeted delivery of ROS nanomaterials to infected lesions, which strongly depends on the physicochemical stability, biological half-life, and tissue-specific targeting capabilities of the nanoplatforms. Moreover, the long-term toxicological risks of ROS nanotechnology, including potential carcinogenicity and teratogenicity, require extensive clinical validation before widespread application. To address these challenges, priority should be given to the development of biodegradable nanomaterials and renally clearable nanoparticles to minimize systemic accumulation and long-term toxicity. Concurrently, image-guided monitoring systems and active-targeting strategies, such as biomimetic membrane coatings or pathogen-specific ligands, provide crucial support for improving delivery prevision and safety during clinical translation of ROS technologies in osteomyelitis treatment 257. Future optimization of ROS-based osteomyelitis therapies should focus on the construction of "triple-function" nanoplatforms that integrate antibacterial ROS generation, immunomodulation, and osteogenic stimulation. In parallel, intelligent closed-loop systems capable of dynamically adjusting ROS production in response to infection biomarkers hold promise for achieving personalized and adaptive therapy. Finally, standardized preclinical models and unified evaluation criteria for antibacterial efficacy will be essential to accelerate regulatory approval and clinical adoption.

The clinical translation of ROS-based technologies for osteomyelitis therapy requires systematic validation across multiple experimental models. Although *in vitro* studies provide critical insights into antibacterial mechanisms, they are insufficient to predict actual *in vivo* antimicrobial efficacy. Small animal models enable preliminary evaluation of immunomodulatory potential; however, their thin cortical bone structure and distinct bone remodeling dynamics do not adequately recapitulate the complex pathophysiology of human osteomyelitis. Consequently, large animal and preclinical studies are indispensable for accelerating the transition of ROS nanotechnology from laboratory research to clinical application. While large animal models offer anatomical and biomechanical relevance, their high cost and limited scalability impede high-throughput evaluation. To overcome these limitations, human-scale bone infection models should be promptly established, incorporating clinically relevant heterogeneity factors like diabetes, vascular insufficiency, and immune dysfunction into experimental design 258. Furthermore, priority should be given to ROS-based technologies that exhibit dual antibacterial and osteogenic effects, thereby maximizing translational efficiency. Emerging new approach methodologies (NAMs) that complement or replace traditional animal testing, including organ-on-chip systems, computational modeling, and advanced imaging techniques, are increasingly applied in osteomyelitis research. Organ-on-chip models can reconstruct interactions between human bone, immune cells, and pathogens, providing high-fidelity evaluation of therapeutic responses at cellular and tissue levels. When combined with human-derived cell-based 3D-printed bone constructs, these systems enable precise testing of local drug delivery strategies and biomaterial performance. Meanwhile, computational models allow simulation of osteomyelitis progression and prediction of treatment effects on infection dynamics, immune responses, and bone regeneration. The integration of NAMs with conventional animal models is expected to substantially enhance experimental reliability, translational relevance, and ethical sustainability, thereby establishing a comprehensive and efficient research framework for the development of ROS-based therapies and next-generation osteomyelitis treatments 259-261.

The clinical application of ROS nanotechnology in osteomyelitis treatment continues to face significant regulatory and ethical challenges. Owing to the unique physicochemical properties and potential biological risks associated with nanomaterials, the establishment of a dedicated regulatory framework is necessary to ensure biosafety, quality control, and standardized manufacturing. Future clinical translation of ROS nanotechnology will therefore require strict standardization of production processes, comprehensive toxicological evaluation, and robust quality assurance systems. Furthermore, the efficacy, safety, and manufacturing consistency of ROS nanomaterials must be systematically benchmarked against existing clinical therapeutics within the same application domains to demonstrate non-inferiority or superiority. Only through rigorous comparative assessment and compliance with regulatory standards can ROS-based therapies achieve reliable, reproducible, and clinically acceptable outcomes, thereby supporting their eventual integration into mainstream osteomyelitis treatment strategies.

## Conclusion

This review comprehensively summarizes the epidemiology, current diagnosis and therapeutic approaches, and pathogenic mechanisms of osteomyelitis, with a particular focus on the evolving role of ROS-based nanotechnologies in its treatment. This review systematically analyzed the development strategies, technical advantages, and inherent limitations of existing ROS-based treatment modalities, including PDT, SDT, CDT, and MWDT, thereby providing an integrative framework to guide the optimization of ROS-driven interventions for osteomyelitis. Furthermore, this review evaluated ROS nanotechnology from multiple functional perspectives—antibacterial activity, immune modulation, and tissue regeneration—and highlighted how the integration of ROS-based strategies with complementary therapeutic modalities can address the multifactorial pathophysiology of osteomyelitis. Such multimodal treatment paradigms offer substantial promise for overcoming the limitations of single-mode therapies and advancing personalized therapeutic solutions. Finally, we critically discussed the remaining challenges of ROS-based technologies, including biosafety concerns, targeting efficiency, mechanistic uncertainties, and translational barriers, while outlining future directions for the development of customized ROS-based therapies tailored to diverse osteomyelitis subtypes and clinical scenarios. Overall, this review provides a panoramic and mechanistically grounded overview of ROS-based osteomyelitis treatment over the past two decades, integrating technological evolution with biological insight. It offers important theoretical practical guidance for advancing our understanding of osteomyelitis pathogenesis and accelerating the development and clinical translation of innovative, next-generation treatment strategies.

## Figures and Tables

**Table 1:** Classification of ROS-based nanotechnologies.

Method	Stimulus	Mechanism	Main ROS types	Advantage	Disadvantage	More suitable type of osteomyelitis	Prospects
PDT	Light of specific wavelength	Type II photochemical reaction	¹O₂	Non-invasive, highly selective, can be applied locally	Limited tissue penetration depth, may cause phototoxicity, requires external light source	Acute/superficial osteomyelitis	New photosensitizers, wireless micro-LED light sources, and integrated diagnosis and treatment platforms
SDT	Ultrasound	US cavitation	OH and ¹O₂	Deep tissue penetration, strong destructive power on biofilms	May cause tissue damage (such as thermal effects or mechanical damage), sonosensitizer delivery challenges	Chronic/biofilm-associated osteomyelitis	Multifunctional sonosensitizer, ultrasound-multimodal imaging guided therapy, portable device
CDT	Microenvironment endogenous substances	Fenton or Fenton-like reaction	·OH	Does not rely on external energy, self-supplies H₂O₂/regulates the microenvironment	Reaction efficiency depends on the local environment (such as pH, H_2_O_2_ concentration)	Acute/chronic osteomyelitis	Intelligent responsive nanocatalytic materials, self-cycling CDT systems, catalytic immunotherapy
MWDT	Microwave	Thermal effect and non-thermal effect	·OH, ¹O₂, or ·O₂⁻	It has strong penetrating ability, rapid treatment, and is effective for deep and encapsulated biofilms	Thermal damage, complex and expensive equipment, difficult control, and the need for precise temperature monitoring	Chronic/deep/biofilm-associated osteomyelitis	Focus on microwave technology, microwave-responsive smart nanomaterials, and real-time ablation tools as surgical assistants
